# Palaeoenvironmental proxies indicate long-term development of agro-pastoralist landscapes in Inner Asian mountains

**DOI:** 10.1038/s41598-021-04546-4

**Published:** 2022-01-11

**Authors:** Michael Spate, Mumtaz A. Yatoo, Dan Penny, Mohammad Ajmal Shah, Alison Betts

**Affiliations:** 1 Department of Archaeology, University of Sydney, Sydney, NSW 2006 Australia; 2 Centre of Central Asian Studies, University of Kashmir, Srinagar, Jammu and Kashmir India; 3 School of Geosciences, University of Sydney, Sydney, NSW 2006 Australia

**Keywords:** Environmental impact, Environmental impact

## Abstract

A growing body of archaeological research on agro-pastoralist populations of the Inner Asian mountains indicates that these groups adapted various systems of mobile herding and cultivation to ecotopes across the region from as early as 5000 BP. It has been argued that these adaptations allowed the development of flexible social-ecological systems well suited to the long-term management of these mountain landscapes. At present, less attention has been paid to examining the long-term ecological legacy of these adaptations within the sedimentary or palaeoenvironmental record. Here we present sediment, palynomorph and charcoal data that we interpret as indicating agro-pastoralist environmental perturbations, taken from three cores at middle and high altitudes in the Kashmir Valley at the southern end of the Inner Asian mountains. Our data indicate spatially and temporally discontinuous patterns of agro-pastoralist land use beginning close to 4000 BP. Periods of intensification of upland herding are often coincident with phases of regional social or environmental change, in particular we find the strongest signals for agro-pastoralism in the environmental record contemporary with regionally arid conditions. These patterns support previous arguments that specialised agro-pastoralist ecologies across the region are well placed to respond to past and future climate deteriorations. Our data indicating long-term co-evolution of humans and landscape in the study area also have implications for the ongoing management of environments generally perceived as “pristine” or “wilderness”.

## Introduction

The Inner Asian mountains comprise a continuous chain from the Altai in the north to the Hindu Kush-Western Himalaya in the south, also arguably incorporating parts of the Tibetan Plateau. The orography of the region creates variability in precipitation patterns, water availability, soil landscapes and vegetation communities that have facilitated a diverse pattern of agro-pastoralist settlement and adaptation from around 5000 BP^1–7^.

Palaeogenomic studies indicate that the development of these agro-pastoral systems was closely linked with movements and admixing of southern, north western, north eastern and indigenous Eurasian populations from ca. 5000 BP onwards^8–10^. Interaction among these populations likely drove the trans-Eurasian exchange of West and East Asian domesticated cereals and legumes including wheat (*Triticum aestivum/durum*), barley (*Hordeum vulgare*), millets (*Panicum* *miliaceum* & *Setaria italica*) and peas (*Pisum sativum*)^3,6,7,11^ as well as livestock including cattle, caprids and horses^12,13^. A growing number of these genetic, archaeobotanical and zooarchaeological studies have lent support to previous conceptions of the Inner Asian Mountain Corridor (IAMC) as a key vector of prehistoric Eurasian “globalisation”^14,15^. It has also been argued that mobility patterns across productive pasture areas of the IAMC were the basis for the development of the historic Silk Roads^16^.

The spread of pastoralist populations across these middle and high-altitude pastures involved not only the movement of novel domesticates into suitable ecotopes, but the modification of foothill and mountain forests into pasture and changes to herbaceous communities within these environmental niches^17^. In this way, pastoralist landscapes across Inner Asia have been characterised as palimpsests of natural and cultural features resulting from the “environmental pragmatism”^18^ of agro-pastoralist societies. In the present day, seasonally mobile forms of cultivation and herding across these landscapes have been described as “multi-resource pastoralism”^19^, comprising a division of labour between herding and other productive industries including farming, resource extraction, or production and trade of secondary goods. This broad and flexible productive base allows for an adaptable society with a high degree of agency in the way they are engaged with pastoralist landscape.

Ethnographic case studies^20,21^ of present day agro-pastoralist groups across the Inner Asian mountains indicate their social-ecological systems developed through long-term processes of landscape evolution and accumulated indigenous knowledge. These flexible systems are well-adapted to ecological management across the region and well positioned to respond to short and long-term climate or environmental variability^20,22^. It has been noted that interventions by colonial administrators, modern governments and other agencies have had detrimental effects on the finely-balanced social-ecological systems these groups inhabit and manage^20,23,24^. As in other parts of the world, we argue that archaeological and palaeoenvironmental studies may make a significant contribution to documenting and preserving these traditional ecological systems, through examining long-term processes of human-landscape interaction in the IAMC^17,24^.

**Figure 1:**
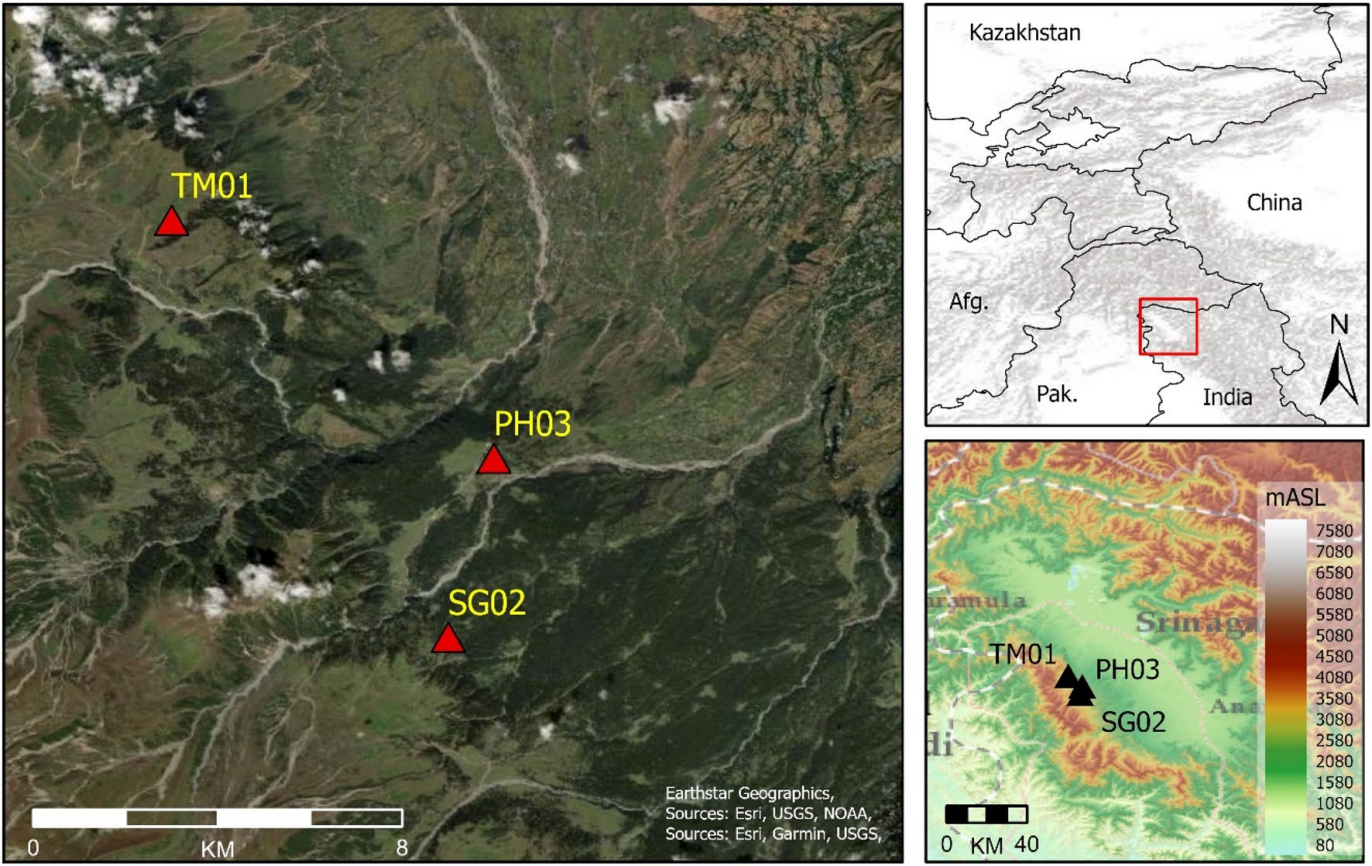
Left) Detail of study area. Top right) location of Kashmir Valley in relation to IAMC. Bottom right) DEM of Kashmir Valley. Base map sources: ESRI. DEM source: ISRO. Images generated in ArcGIS Pro v2.7.0 (https://www.arcgis.com/).

The co-evolution of environments and pastoralist populations across Inner Asia has been viewed through theoretical lenses such as Niche Construction Theory or Ecosystem Engineering, which aim to describe anthropogenic perturbations and feedback loops between environments and pastoralist societies, primarily through on-site archaeobotanical, zooarchaeological and geoarchaeological studies aimed at examining herbaceous communities, grazing and cultivation patterns and soil formation processes^17,24,25^. By recognising present-day mountain pastures as partially anthropic landscapes, we argue that their development may also be observed through changes in palaeoenvironmental data. Here we present a multi-proxy study of three sediment cores sampled at middle and high-altitude pastures in the Kashmir Valley, at the southern end of the IAMC (Fig. 1). Sample sites are all located within a present-day landscape dominated by mixed cultivation and herding land use, at elevations between 2600 and 3100 m ASL (Figs. S1–S4).

One case study^26^ on grazing social-ecology in the Kashmir Valley describes the utilisation and modification of various altitudinally differentiated ecological niches during the seasonal migration cycle of Bakarwal pastoralists. On the Pir Panjal flank of the Kashmir basin, pastoralists clear areas of *Pinus* forest and suppress growth of saplings, opening the wooded areas into new ecological niches. These newly opened landscapes are colonised by various members of the *Polygonum, Rumex*, *Chenopodium*, Caryophyllaceae, Fabaceae and Asteraceae genera and families, either through deliberate propagation as favoured herbs or as commensal spread associated with large groups of herbivores. Several studies on the ecological impacts of grazing^27–29^ also found that moderate grazing may enrich herbaceous diversity of pastures, while declining diversity and an overabundance of Poaceae as well as high proportions of unpalatable *Urtica*, *Plantago* and Asteraceae types were associated with overgrazing. These studies also found high representation of *Rumex, Polygonum* and *Trifiolium* in heavily grazed meadows, colonising nitrogen-enriched areas of dung accumulation. The relationship between pastoralism and geochemical and sedimentary change has also been studied in meadows on the mountain flanks in Kashmir^30^. This study found a linear relationship between nitrogen, potassium, phosphorus and sulphur, all increasing on a gradient from lightly to heavily grazed areas. In addition, significant increases in the coarse sand fraction of surface sediments was recorded in relation to heavier grazing. These observations provide an interpretive basis for the palaeoenvironmental proxies we present here.

## Results

**Figure 2:**
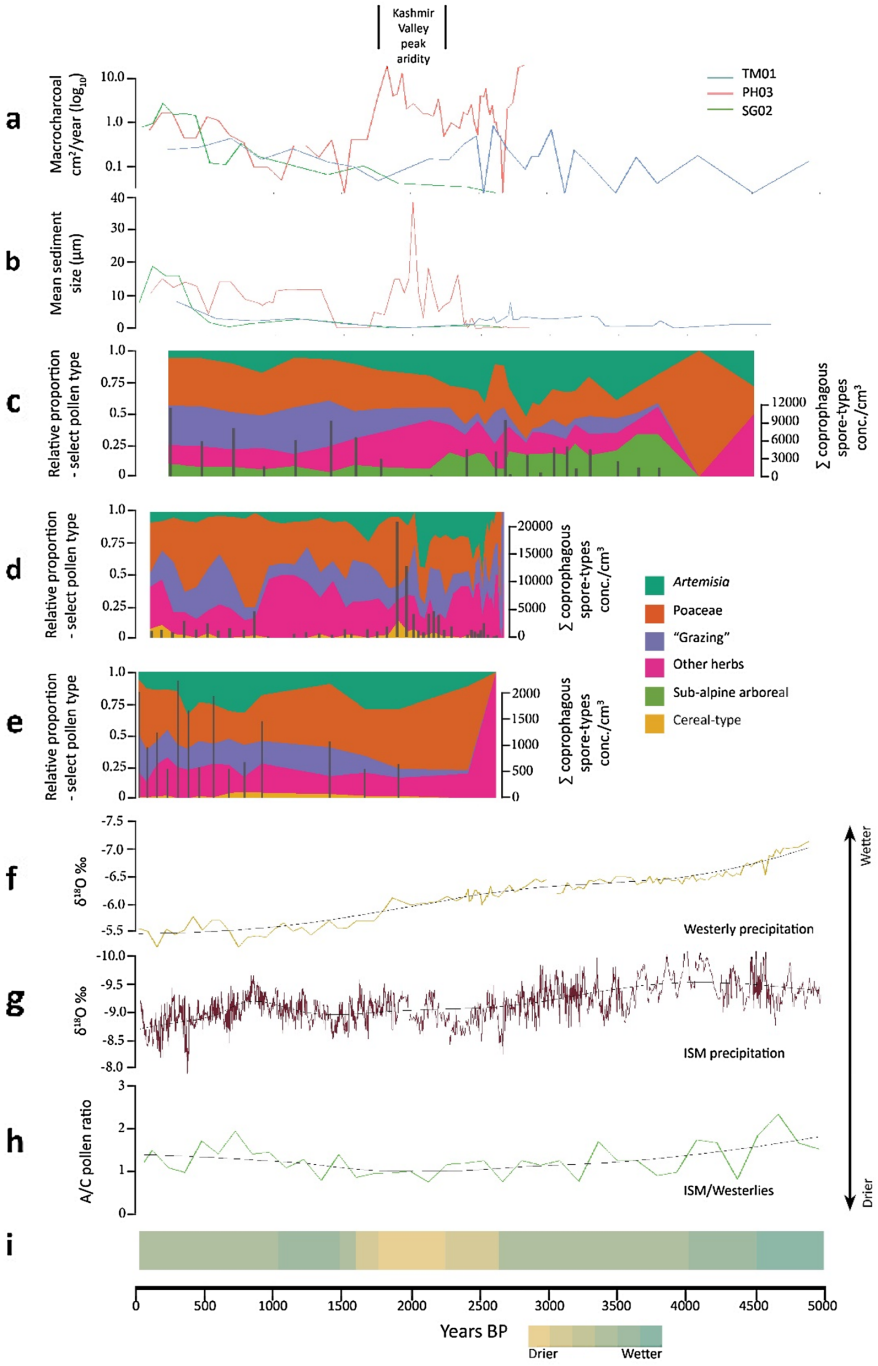
Proxy data related to agro-pastoralist land use from this study: (**a**) Macrocharcoal annual influxes; (**b**) Mean mineral sediment size; (**c**) TM01, (**d**) PH03 and (**e**) SG02 relative proportion of pollen types and summed coprophagous spore concentrations. (**f**) δ^18^O record, Ton Cave, Uzbekistan^46^. (**g**) δ^18^O record, Sahiya Cave, India^47^. (**h**) A/C pollen ratio, (lake) Tso Moriri, Ladakh^57^. (**i**) Inferred environmental conditions, multiple records, Kashmir Valley^43,54,55^.

Our review of relevant literature on grazing ecology and impacts, as well as in-field observations, identified environmental changes associated with pastoralist land use and modification and their associated proxies within the sedimentary record (Table S1; Fig. S4). A total of 39,207 identified pollen grains and fern spores were counted across the three sediment cores. These pollen data have been plotted along with fungal spore concentration, charcoal influx and mineral particle size data in Figs. S5–S10. Radiocarbon dates and age depth models indicate a temporal coverage of ca. 5500 years in the TM01 record, ca. 2800 years in the PH03 record and ca. 2700 years in the SG02 record (Figs. S5–7). Results here (Fig. 2 a-e) are divided chronologically, following established archaeological or historical periods in Kashmir^31,32^. Principal component analysis found correlation between proxies we associate with agro-pastoralism across all three records (Figs. S11–S14).

The TM01 record partially spans the prehistoric Neolithic period in the Kashmir Valley (ca. 4000–3000 BP; 96-70 cm depth in core) and during this period the sedimentary record at the site contains low levels of indicators of pastoralist land use, including summed coprophagous spore types averaging 837/cm^−3^, 7% mean relative abundance of pollen types associated with grazing and relatively coarse, very poorly sorted mineral sediment (mean size 2.4 µm, mean S_g_ 6.12).

During the first few centuries of the Proto-historic/Iron Age period (ca. 3000–2000 BP) indicators of pastoralist land use in the TM01 record fluctuate, with peaks in grass (Poaceae) pollen (15.5% & 15.8% relative abundance), coprophagous spores, and mean sediment size (two peaks at 7.9 µm and 3.7 µm) in the centuries either side of 2500 BP. At ca. 2600 BP is a peak in > 250 µm (0.378 particles/cm^2^/year) and 125–250 µm (0.5 particles/cm^2^/year) size classes of charcoal. At ca. 2300 BP, *Artemisia* pollen begins a steady decline, as do sub-alpine arboreal types (summed *Betula, Salix, Juniperus, Alnus* and *Corylus* types) from ca. 2200 BP. A more rapid sediment accumulation rate in the PH03 record/core allows a better sampling resolution that reveals frequent, small-amplitude peaks in charcoal, coprophagous spores (mean 405/cm^3^) and low levels of cereal-type pollen (0–0.8% relative abundance) between ca. 2600 and 2450 BP (300–275 cm depth in core). Between ca. 2200 and 2000 BP (225–185 cm depth in core), there are increases in cereal pollen (0.3–1.5%) relative abundances of herbaceous pollen types suggestive of grazing (mean 6.8%), minor peaks in both charcoal size fractions (2.86 and 28,682 particles/cm^2^/year), and *Podospora*-type (2245/cm^−3^) and *Sordaria*-type (2570/cm^3^) fungal spores.

In the PH03 record between ca. 2000 and 1800 BP (185–145 cm depth in core) peaks in several variables including Poaceae (to 17% relative abundance), pollen types associated with grazing (to 10% relative abundance), macro-charcoal (20 particles/cm^2^/year) and summed coprophagous spores (to 21,884/cm^3^) coincide with sharp declines in *Artemisia* pollen. During this period cereal pollen types compose up to 5% of the overall assemblage. Very poorly sorted (σ*G* > 6) and relatively coarse (mean size 18 µm) mineral sediment is deposited through this period. Pollen indicators of grazing in the TM01 core increase from 6 to 20% relative abundance between ca. 2000 and 1400 BP (32–24 cm depth in core), and summed coprophagous spore types, absent from the record at 2200 BP, increase sharply to 9360/cm^3^ by 1400 BP. The relative abundance of Poaceae pollen gradually increases while *Artemisia* declines. The relatively slow sediment accumulation rate during this period reduces temporal resolution, but all proxies indicate a protracted 600 year-long increase of intensifying pastoralist land use around the site. Mean mineral particle size increases steadily through this period, from a minimum of 0.3 μm at ca. 2100 BP (30 cm depth in core). Indicators of pastoralist land use around both sites are contemporary with the Kushan period in the Kashmir Valley, ca. 2000–1500 BP.

In the PH03 record there is a sharp reduction in all proxies associated with pastoralist activity between ca. 1500 and 800 BP (135–100 cm depth in core), though it is unclear if this is the result of masking by stronger environmental signatures associated with a lithostratigraphic shift from humified mud/peat to clay bedding. The same period sees a slight decline in concentrations of *Podospora* and *Sporormiella*-type spores in the TM01 record (32–16 cm depth in core), possibly suggesting a reduction in grazing activity in the area, while macro-charcoal influxes remain below the long-term average. Grazing-related pollen taxa (*Rumex,* cf. *Trifolium*, Caryophyllaceae) remain at stable levels through this period, while taxa indicating higher levels of disturbance (*Plantago*, *Urtica*) are present at very low, fluctuating levels.

From 800 BP to present, proxies associated with pastoralist activity begin to increase in both cores. In the PH03 record (100–1 cm depth in core), increased concentrations of *Podospora* (mean 698/cm^3^) and *Sordaria*-type (mean 1008/cm^3^) spores, an increase in the relative abundance of *Rumex* (0.5–3.3%), *Plantago* (0.1–2.3%), *Urtica* (0–1.9%), Poaceae (3.6–20%) and cereal-type pollen (0–1.6%), and the deposition of coarse, very poorly-sorted sediment (sand 3–30%, mean σ_*G*_ 7.6) may relate to erosional processes associated with land clearing and consequent colluvial mobilisation around the site. The abundance of *Artemisia* pollen declines to the lowest levels (mean 0.89%) in the entire sequence during this period. In the TM01 (15–1 cm depth in core) record, there is a sharp increase in the proportion of sand-sized mineral clasts (6–29%) and the particle size distribution is very poorly sorted (σ_G_ = 5.9–9). Due to the slow accumulation rate in the upper section of the TM01 record, and the rejection of a modern AMS date (D-AMS 034134) at 23 cm depth in core, we caution against over interpretation of variability throuhg this period, however multiple proxies indicate strong signals for pastoralist land use.

The SG02 record presents the strongest evidence for the impact of pastoralists on the landscape from ca. 500 BP to present (55–1 cm depth in core). During this period pollen associated with grazing increases (3.5–11%), summed coprophagous spore types increase to an average of 1374/cm^3^, and particle size data is dominated by coarse (sand fraction 5–33%) and very poorly sorted (mean σ_*G*_ 11) sediment.

## Discussion

### Temporal and altitudinal variation in land use and modification

**Figure 3:**
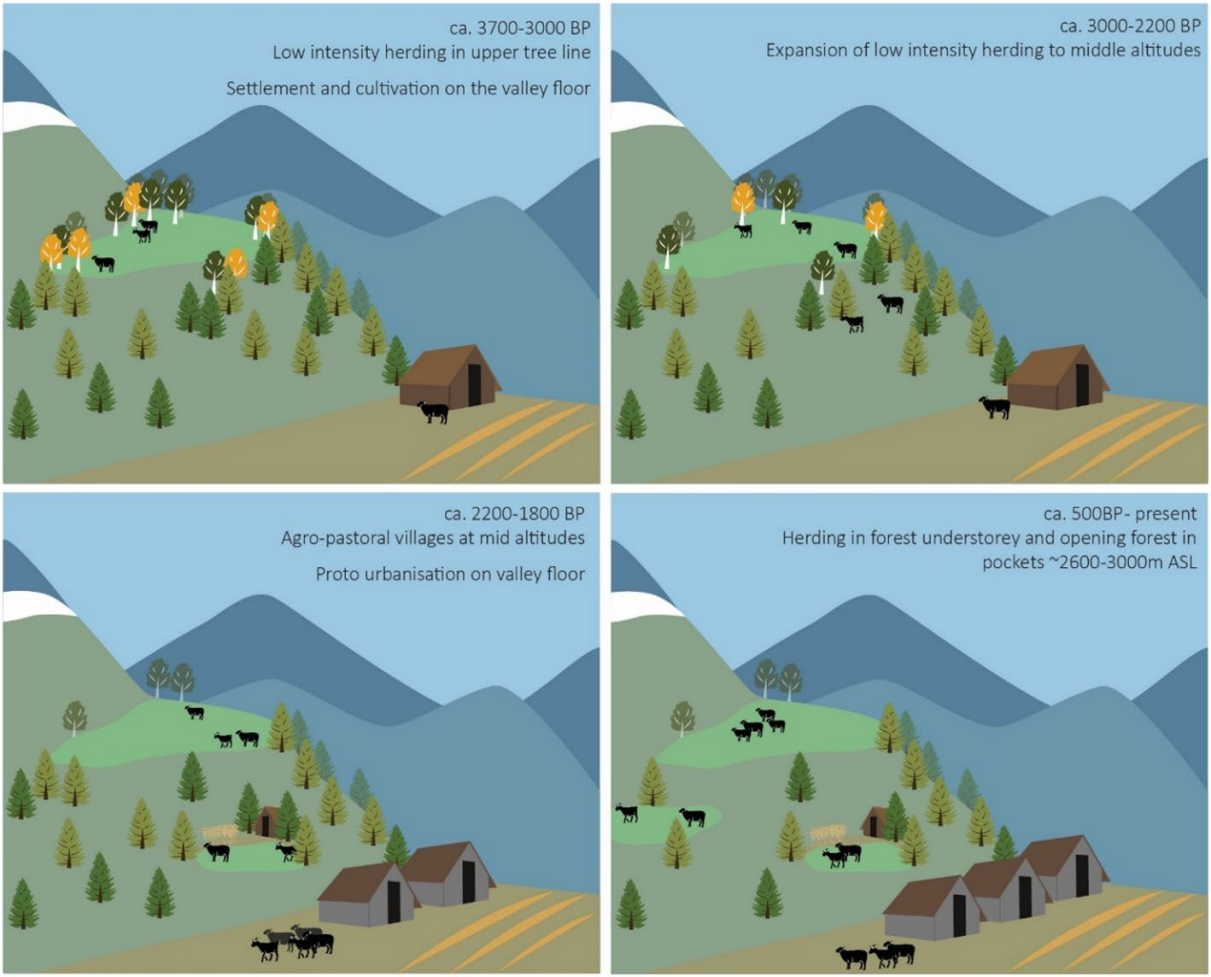
Temporal changes in pastoralist land use pattern as interpreted from study data. Image generated in Adobe Illustrator v25.4.1 (https://www.adobe.com/au/products/illustrator.html).

At the high altitude (3100 m ASL) Tosa Maidan site (TM01) there appear to be three stages of intensified pastoralist activity; ca. 3700–3000 BP, 2700–2500 BP, and 2000 BP-present. The period ca.3700–3000 BP exhibits a low-level of perturbation, where moderate levels of palatable herbs such as *Rumex* and Caryophyllaceae were present. During this period there does not appear to be heavy pressure on sensitive taxa such as *Artemisia,* and levels of Poaceae generally remain below 20% of the total pollen assemblage. A slight decline in sub-alpine arboreal/shrub pollens may be evidence of low levels of clearing, which is corroborated by increased influx of macro-charcoal and coarse sediment reflecting local burning and sediment mobilisation. These perturbations in the record fall within the Late Neolithic and Megalithic archaeological phases in the Kashmir Valley^31^, where known settlement patterns are typically agricultural villages distributed on the valley flanks between ca. 1600 and 2000 m ASL^33^. A number of Neolithic settlement sites have been reported from Budgam District, to elevations of around 2100 m and within 10 km of Tosa Maidan^34^. Published zooarchaeological data from these Late Neolithic^35^ sites indicate a shift from mixed wild/domestic to almost completely domestic animal assemblages through this period. Against this background, the perturbations associated with herding activities in the TM01 record may be interpreted as low investment summer herding around the tree line (Fig. 3). This broadens the previously understood range of land usage in Kashmir during this period.

Between ca. 2700 and 2500 BP it appears that proximal land use began to impact this high-altitude ecological niche. Caryophyllaceae and *Artemisia* decline and relative increases of *Plantago, Urtica,* Poaceae and Asteraceae possibly indicate intensive grazing, or overgrazing. A sharp increase in mean mineral particle size, an increase in the influx of macro-charcoal size classes, and a marked decline in the abundance of *Betula* pollen also point to intensified land-clearing. Diversity of plant taxa recovers after this period until ca.2000 BP, after which there is a steadily increasing trend for indicators of grazing. Throughout this period the ecological niche appears to be maintained through suppression of high-altitude trees and shrubs such as *Betula, Abies* and *Juniperus,* as well as episodes of less intensive land use. For example, between ca. 1000 and 800 BP the recovery of *Artemisia* is coincident with a decrease in coprophagous spores.

Evidence for niche constructing activities at Pari Has (PH03) take on a slightly different character due to the site’s lower elevation and surrounding coniferous forest cover. A large influx of all charcoal-size classes from the start of the record at ca. 2700 BP may be interpreted as human-induced burning of the forest landscape, contemporary with a period of intensive land use in the TM01 record. Detecting anthropogenic opening of the landscape remains problematic as pollen from conifers are likely over-represented in local pollen fallout^36,37^. However, several taxa in the pollen assemblage—such as *Plantago* and Caryophyllaceae—are from plants with photosynthetic requirements for an open environment^38^. Surface studies of modern pollen in the Western Himalayas also indicate that herbaceous types are generally more representative of vegetation around the sample site^36^. The data in the PH03 core may, therefore, indicate a transition from a more closed forest canopy to a more open environment between ca. 2300 and 2100 BP (Fig. 3). *Urtica* and Cannabaceae pollen are also evidence for disturbance of the landscape in this period, as is an increase in mean sediment particle size.

Evidence of agro-pastoralist land use from mountain areas for the period 3000–2000 BP in Kashmir is significant, since archaeological data for settlement at lower elevations are poorly documented relative to the preceding Neolithic period^39^. Based on preliminary survey data, it has been argued that this period saw a shift of economic focus away from the valley floor and into middle-altitude areas^40^. The data presented here support these interpretations and are also consistent with a diversification of social-ecological strategies including increased mobile herding across the IAMC during this period^41^.

Between ca. 2000 and 1800 cereal-type pollen, coprophagous fungal spores, pollen indicators of grazing, charcoal influx and mean particle size increase sharply in the PH03 record, indicating intensified agro-pastoralist signals around Pari Has. These data are contemporary with the early Kushan Period in the Kashmir Valley, a time of major social reorganisation and the integration of Kashmir into regional polities^42^. This period is also noted for the increased distribution of settlements across the valley, as well as their expansion into proto-urban towns^42^. The data in the PH03 record indicates significant changes in land use around this middle altitude site and may be evidence of the initial movement of agro-pastoral villages and cultivation to these elevations.

The decline of proxies for pastoralism and cultivation around Pari Has between ca. 1500 and 800 BP can be interpreted as a response to the development of wetter local environmental conditions that rendered the site less suitable for agro-pastoralism^43^. The site currently sits at the altitudinal limit of year-long occupation in Kashmir and a shift to cold-wet conditions would reduce the suitability of this type of land use. Additionally, it is likely that wetter conditions drove enhanced surface runoff that led to a focusing of cultivation on the valley floor. This second interpretation is tentatively supported in the archaeobotanical record, which indicates a diversification and expansion of both winter and summer agriculture^44^. In particular, rice becomes the numerically dominant crop among archaeobotanical assemblages^35,44^, and we hypothesise that higher water availability may have allowed the development of the systems of rice paddy agriculture practiced in the valley today. This concentration of paddy labour may have been associated with other processes of state formation, urbanisation and centralisation of bureaucracy taking place in Kashmir at this time^32,45^. During this period our data from the high-altitude Tosa Maidan site indicate weak and fluctuating signals for pastoralist land use, implying that the pastoralist landscapes were either dispersed over a wider geographic space, or there was a shift to lower investment herding close to the timber line, where suppression of forest growth and maintenance of the pastoralist niche would have been less labour intensive.

Evidence for pastoralist land use around Shali Ganga (SG02) occurs much later than that around Pari Has, with significant evidence only after ca. 500 BP in the form of increased mean mineral sediment size, influxes of charcoal and high concentrations of coprophagous spores. Despite the relative proximity of the site to Pari Has, the lack of the economic or environmental imperative to make use of this part of the landscape, in addition to constraints imposed by the topography of the area, may have been limiting factors. Indicators of pastoralist or agro-pastoralist land use are discontinuous within records and changes are often asynchronous between the records. Comparing these data, it is possible to interpret a dispersed or “patchy”^17^ pastoralist landscape across the district. Sheltered niches such as Pari Has, while suitable for enrichment and cultivation during favourable environmental conditions, could have been increasingly difficult to maintain when the labour investment could be productively directed elsewhere. The evidence for grazing and clearing of understory at SG02 during the last 500 years may result from several social, demographic or environmental factors that drove the utilisation of this more marginal niche. This variability can be compared with observed agro-pastoral use at the present day, where groups of herders move to the area from the lower altitudes of the region at the start of summer. Depending on environmental conditions, strategic decisions are made as to whether to remain in the area and engage in cultivation of summer crops (typically maize), move the herds to higher altitude sites such as Tosa Maidan, or undertake a division of labour to engage in a mixture of these two choices. These strategies comprise a flexible social-ecological system that can respond to changing environmental and historical circumstances at annual or even seasonal scales. The data presented here indicate that these social-ecological systems are long-standing adaptations, and the accumulation of thousands of years of agro-pastoralist productive strategies may be detected among the accrued changes in the palynomorph, sediment and charcoal records.

### Responses to Holocene climate variability

Speleothem δ^18^O data from the Himalayas and Inner Asian mountains generally indicate weakening winter Westerly (Fig. 2f) and Indian Summer Monsoon (Fig. 2 g) precipitation between ca. 4000 and 2000 BP^46–49^. Despite localised variability among regional lake or sediment records, multiple proxies indicate an overall drier environment during this period^50–53^. Environmental indicators from the current study sites^43^, other Kashmir Valley records^54,55^ (Fig. 2i) and proximal lake records from Ladakh (Fig. 2h)^56,57^ indicate maximally arid conditions ca. 2200–1800 BP, following a period of apparent instability indicated by fluctuating wet/dry conditions ca. 2700–2500 BP^43,54^.

Based on modelled climate change data^58^ it has been argued that regional climate shifts stimulated the exchange between and diversification of agricultural packages between 4000–2000 BP. These processes may be seen in archaeobotanical and archaeological evidence for the spread of diverse cropping, irrigation and settlement systems across a number of altitudinally varied ecotopes in Inner Asia, particularly between ca. 3000–2000 BP^41,59,60^. Given indicators for agro-pastoralist land use in the PH03 and TM01 during drier environmental phases, we argue that an intensified focus on herding in middle and high-altitude regions may have been an adaptive strategy to mitigate the effects of protracted aridity at lower elevations. This emphasis on upland herding may be seen again after ca. 1100 BP, where it has previously been hypothesised, based on tree ring data for weaker Westerly precipitation^61^, that aridity was a driver of a shift to more mobile forms of herding across the Western Himalayas. The indicators for grazing and cultivation in the PH03 record from ca. 1100 BP and grazing in the SG02 record after ca. 500 BP may reflect the focusing of economic activity to middle altitude areas and marginal forest niches as a response to these environmental conditions.

## Conclusion

This study presents evidence for temporally and spatially varied land use across an altitudinal gradient from the Kashmir Valley. Our data indicate that the land use patterns of present day agro-pastoralists in the area have an ecological heritage stretching back over 3500 years. We expect to see these patterns of land use repeated across the Inner Asian mountains, where the movements and adaptations of ancient agro-pastoral populations not only drove significant interaction between Eurasian populations but also transformed landscapes and produced anthropic environmental niches. These long-term human–environment interactions would indicate that well-managed pastoralist social-ecological systems have been closely adapted to Holocene climate and environmental perturbations and are central to maintaining a resilient ecosystem across the region today.

## Materials and methods

Targeted coring sites were mires or swamps located in pasture areas at middle or high altitudes of Budgam District on the western flank of the Kashmir Valley (Jammu & Kashmir, India) (Figs. S1–3). Sediment cores were sampled using a D-section peat corer manufactured by Dormer Australia (inner ø50mm; chamber length 500 mm). Sediments were sampled in overlapping 50 cm sections until refusal on compact bedding structures.

Accelerator Mass Spectroscopy (AMS) ages were measured on plant material (twigs, wood), charcoal, and bulk organic samples (supplement). Samples were treated with 10% KOH at the University of Sydney, dried then pre-treated with standard acid–base-acid procedures and dated at DirectAMS, Seattle. Returned dates were calibrated in Calib v8.02^62^ with the Intcal 20 calibration curve^63^ (Table S2). An age depth model was produced using the Bacon (v2.5.7) package in R^64,65^. All prior settings were left as default unless otherwise described in Figs. S5–7.

1 cm^3^ sediment subsamples were oxidised and disaggregated following previously described procedures^43^. Particle size distribution was measured by laser diffraction using a Malvern Mastersizer 2000 with Hydro dispersion attachment (Figs. S5–7). Sediment subsamples were taken at depth intervals equivalent to < 100-year temporal resolution wherever possible. Charcoal and palynomorphs were extracted and identified following procedure in Spate et al.^43^ and plotted in Tilia v3.0.1 (Figs. S8–10). A minimum target of 150 individual non-arboreal pollen was set for each slide, except for samples where pollen concentrations were extremely low. Concentrations/cm^3^ of coprophagous spores were calculated using the dilution of a known number of *Lycopodium* spores introduced to the sample during pre-treatment as an artificial spike (Lund University batch #1031; total spores 20,848 ± 691). Annual influxes/cm^2^ of macro-charcoal were calculated using accumulation rates from the age-depth model. Structures in the data were explored using Principal Components Analysis (PCA, Figs. S11–14) in PAST v3.17^66^ following standardisation of variables by subtracting the mean from each observation and dividing by the standard deviation^67^.

## Supplementary Information

Supplementary Information.

## Data Availability

Raw and processed data for this study, pollen/spore identification criteria and photographic plates have been placed in a repository and are available at https://datadryad.org/stash/dataset/doi:10.5061%2Fdryad.wdbrv15nb.
