# Supplementary material for: Palaeoenvironmental proxies indicate long-term development of agro-pastoralist landscapes in Inner Asian mountains
Source: Sci Rep. 2022 Jan 11;12:554. doi: 10.1038/s41598-021-04546-4 (PMC8752612; doi:10.1038/s41598-021-04546-4)
Supplement: Supplementary file 1 — Supplementary Information. [file 41598_2021_4546_MOESM1_ESM.pdf]

Supplement to:

Palaeoenvironmental proxies indicate long-term development of agro-pastoralist landscapes in Inner Asian mountains.

Michael Spate [a,] *, Mumtaz A. Yatoo [b], Dan Penny [c], M. Ajmal Shah [b], Alison Betts [a]

[a] Department of Archaeology, University of Sydney, NSW 2006, Australia

[b] Centre of Central Asian Studies, University of Kashmir, Srinagar, India

[c] School of Geosciences, University of Sydney, NSW 2006, Australia

* Corresponding author: michael.spate@sydney.edu.au

Materials and Methods

Fieldwork and sample collection: Study sites are all located in Budgam District (Jammu & Kashmir, India), on the western Pir Panjal flank of the Kashmir Valley, between 74.4533 and 74.8073 E (DD), and 34.1385 and 33.8100 N (DD). The district contains a varied topography, from the Jhelum alluvial zone at ca. 1600m ASL to high peaks over 4000m ASL. Several large, well-known meadows in alpine and sub-alpine zones include Doodhpathri, Yusmarg and Tosa Maidan. Below the tree line, these meadows are interspersed through conifer forests. At some larger pastures such as Yusmarg, Gujjar pastoralists build semi-permanent villages as a base for herding to higher altitudes, while part of the community engages in cultivation of maize and other crops as well as guiding and trekking in the tourism industry. Agricultural villages are present up to an altitude of around 2400m ASL. Kashmiri farmers in these villages generally engage in a mixture of horticulture, cultivation of summer vegetables and cereal, as well as transhumant herding to higher pastures throughout the district. Fieldwork site selection follows the assumption that pasture landscapes are at least partially of anthropic origin. Sediment cores samples from mires or lake/swamp margins are generally more suitable for examining human-induced changes to pollen spectra than larger lakes [1,2] and were targeted here for exploring the development of these pastoralist landscapes.

Tosa Maidan – core TM01

Tosa Maidan (74.518802 E, 33.921172 N) is a large sub-alpine meadow at around 3100m ASL, within a glacially excavated basin (**Figure S1**). Preliminary works on post-glacial vegetation successions were undertaken by Gurdip Singh [3], with a preliminarily dated to ca. 18000 BP [4]. The basin is ringed by high peaks to the west, with lower rising slopes to the north and south. The north-eastern side of Tosa Maidan rises steeply before falling away more sharply towards the Kashmir Valley basin. The Sokhnag River skirts south of the basin, with several streams across the meadow draining into the river. The slopes surrounding the basin are covered in *Abies-Cedrus* stands that are currently extensively logged towards the east. Areas of sub-alpine *Juniperus* scrub were also observed. Open area land cover is typical meadow vegetation, including various genera from the Poaceae, Asteraceae and Polygonaceae under moderate grazing during summer 2018. Al-Biruni noted Tosa Maidan as an important pass into the Kashmir Valley during the 11th century and remained the route

of military incursions into Kashmir as late as the 19th century [5]. At present, Tosa Maidan is grazed during summer by flocks of sheep and goats, either herded from proximal villages from the district for short periods of time or penned in association with seasonally inhabited structures for longer durations. Coring site TM01 is a marshy area on the southern flank of the basin, on a terrace below fir-covered slopes. Sediments appear to accumulate through in-wash from the slopes above, with gentle drainage outflowing to the floor of the basin to the southwest. D-section coring reached a depth of 130cm, where a compact clastic bed was impassable.

Shali Ganga - core SG02

Coring site SG02 (74.572713 E, 33.8345456 N) is located in an open grazed areas on a rise above the Shali Ganga river in the Doodpathri area at 2880 m ASL. The coring site and the surrounding landscape are a mosaic of open, semi-open and closed conifer forest areas that are subject to varying intensities of grazing. Semi-permanent pastoralist settlements are in open areas a few hundred metres from the coring site (**Figure S2**). D-section coring was undertaken to a depth of 1.15m, until refusal on a heavily compact blue-grey clastic bed.

Pari Has – core PH03

Pari Has (74.581588 E, 33.874805 N) is a swamp located in a basin in the Doodhpathri area, situated around 2km west of the farming village of Erzil, at an elevation around 2600m ASL. Sediment accumulation at the site takes place through downslope in-wash from a series of slopes and terraces to the west and south (**Figure S3**). A series of low undulating rises have impounded drainage, leading to the development of swampy conditions though some gentle outflow takes place to the northeast. The sharpest slope to the west rises to an expansive meadow area known as Danger Pora, currently under heavy grazing as well as recreational usage. The open areas and forest understory around the coring site are grazed daily, with herds of sheep penned close by then released during the day. The area is typically inaccessible in winter, however, there are small, semi-permanent settlements throughout the Doodhpathri area where pastoralists reside during summer as a base for herding as well as secondary economic activities such as small-scale forestry. *Abies pindrow* and *Pinus wallichiana* are the dominant coniferous stands. Herbaceous ground cover is dominated by grasses, sedges, *Trifolium* and *Potentilla* mats. Heavily disturbed areas are colonised by *Circium*, *Urtica*, *Plantago*, *Viburnum* and *Sambucus*. Core PH03 was taken using a D-section corer to a depth of 3.55m where a compact grey-blue clay resisted further sampling.

Proxy indicators for pastoralist land use in Kashmir: Casimir & Rao [6] have described the utilisation and modification of various altitudinally differentiated ecological niches in Kashmir and adjacent areas during a seasonal migration cycle of Bakarwal pastoralists. On the Pir Panjal rim of the Kashmir basin, pastoralists clear areas of *Pinus* forest and suppress growth of saplings, opening the wooded areas into new ecological niches:

"There is good pasture on the northern slopes of the Pir Panjal at an altitude of about 2,500m in the *Cedrus-Pinus* formation …On the whole, the undergrowth of the woodlands and their marginal zones is characterized by dense stands of *Gallium, Geranium, Trigonella, Astralagus, Potentilla, Euphrasia, Tanacetum, Alchimila*, and the genera *Polygonum, Chenopodium, Portulaca*, and *Cerastium*, all gathered intensively by the Bakrwal and eaten as vegetables. The fields, cut as wide aisles through the woods, serve as pasture." [6].

Given the relatively high pollen productivity and dispersal of conifer pollen in the Western Himalayas [7], detecting deforestation among in palaeo-records from these forest belts remains complicated. We argue that pastoralist impacts to the landscape may be better detected through the suppression of taxa noted for their sensitivity to grazing, notably *Artemisia* [8], as well as increases herbaceous pollens associated with open, grazed landscapes.

Studies [9–13] on the impact of grazing on herbaceous diversity generally found that moderate grazing may enrich niche diversity, while declining diversity and an overabundance of Poaceae as well as high proportions of unpalatable *Urtica*, *Plantago* and Asteraceae types were associated with overgrazing. These studies also found high representation of *Rumex, Polygonum* and *Trifiolium* in heavily grazed meadows, colonising nitrogen-enriched areas of dung accumulation. The relationship between pastoralism and geochemical and sedimentary change has also been studied in meadows on the mountain flanks in Kashmir [14]. In addition to the nitrogen enrichment, this study found a linear relationship between nitrogen, potassium, phosphorus, and sulphur, all increasing on a gradient from lightly to heavily grazed areas. The coarse sand fraction of surface sediments also increased from 4.2±1.7% in lightly grazed areas to 13.7±4.16% at sites of heavy grazing.

The findings from these studies are consistent with our own observations of the impacts on varying intensities of grazing on herbaceous vegetation communities and ground cover condition around our study sites (**Figure S4**). These descriptions allow the identification of proxy indicators for examining pastoralist impacts on the landscape in palaeoenvironmental records. More generalised studies of other proxies include charcoal influxes [15] as evidence of human-induced burning, or coprophagous spores [16] as indicating large numbers of herbivores in the study area. Variability of individual proxies to may be driven by several factors including climate change or other human-induced or natural processes. The co-variation of multiple proxies, particularly a combination of biotic (pollen, fungal spores, charcoal) and abiotic (sediment) types may allow for a stronger interpretation that these variations were a result of pastoralist activity, particularly when these have different depositional pathways into the palaeoenvironmental record.

Table S1: Environmental proxies associated with agro-pastoralism

**Proxy**	**Type**	**Description**	**Reference**
Poaceae	Pollen	Increased grass cover and lower herbaceous diversity has statistical relationship with grazing intensity	9–11
Poaceae – cereal type (above 38µm)	Pollen	Cultivation associated with agro-pastoralism	-
Rumex, Caryophyllaceae, *Trifolium*-types	Pollen	Plant types associated with nitrogen enrichment of grazed areas. Forage herbs for human and animal consumption. *Rumex* often absent from ungrazed/undisturbed areas	6,10,17
Asteraceae, *Plantago*, *Urtica, Sambucus*	Pollen	Ruderal and unpalatable plants associated with degraded landscapes and over grazing.	9–11

Proxy	Type	Description	Reference
Artemisia	Pollen	Open landscape shrub, highly sensitive to grazing pressures. Declining values related to presence of herbivores.	[8,10]
Podospora, Sordaria, Sporormiella-types	Fungal spores	Coprophagous spores associated with herbivore dung. Associated with summer grazing ruminants in Himalayan region	[16,18]
Mean particle size, sorting values	Sediment	Larger, more poorly sorted sediment may relate to herbivore or human induced erosion.	[14]
Macro-charcoal (125-250 µm & >250µm)	Charcoal	May be evidence of localised anthropogenic burning – forest clearing or campsites.	-

Table S2: AMS dates from sediment cores. Calibrated in Calib 8.2, Intcal20 curve [19,20]

Core	Sample code	Lab code	Material	Depth (cm)	^{14}C age BP	1σ range (cal. BP)	% of probability distribution
**TM01**	TM01_1.1_23	D-AMS 034134	Bulk organic	23	36 ± 26	N/A	N/A
	TM01_1.1_33	D-AMS 034135	Bulk organic	33	2554 ± 29	2543 - 2558<br>2618 - 2630<br>2703 - 2744	14.5<br>12.5<br>72.9
	TM01_1.1_40	D-AMS 032554	Bulk organic	40	2708 ± 28	2764 - 2792<br>2818 - 2846	52.5<br>47.5
	TM01_2.1_61	D-AMS 032555	Bulk organic	61	2618 ± 30	2737 - 2757	100
	TM01_2.1_77	D-AMS 034136	Bulk organic	77	2937 ± 29	3008 - 3012<br>3059 - 3161	28<br>97.2
	TM01_2.1_88	D-AMS 031622	Bulk organic	88	3443 ± 35	3729 - 3742<br>3773 - 3791<br>3826 - 3897	8.5<br>15.1<br>76.3
**PH03**	PH01-A1	D-AMS 023859	Peat	95	832 ± 26	689 - 713<br>716 - 739<br>763 - 770	42.9<br>47.8<br>9.3
	PH01-C1	D-AMS 023860	Peat	135	1948 ± 49	1829 - 1859<br>1869 - 1892<br>1905 - 1922	39.7<br>40.7<br>19.7
	PH03_1.3_178	D-AMS 032549	Twig	178	2102 ± 29	2003 - 2035<br>2042 - 2019	23.7<br>75.3
	PH03_2.3_245	D-AMS 031342	Twig	245	2079 ± 27	1995 - 2062<br>2084 - 2098	86.9<br>13.1
	PH03_2.4_295	D-AMS 032553	Bulk organic	295	2564 ± 24	2548 - 2551<br>2621 - 2627<br>2708 - 2748	2.7<br>7.1<br>9
	PH03_1.2_332	D-AMS 031343	Twig	332	2424 ± 30	2363 - 2472<br>2474 - 2489<br>2605 - 2609<br>2661 - 2664	84.1<br>11.1<br>2.8<br>2
**SG02**	SG02_1.1_48	D-AMS 032550	Wood	48	347 ± 23	315 - 411<br>421 - 477	61.4<br>38.6
	SG02_2.1_79	D-AMS 032551	Wood	79	919 ± 26	746 - 760<br>771 - 913	4.6<br>95.4
	SG02_1.2_105	D-AMS 031340	Charcoal	105	2491 ± 31	2434 - 2450<br>2462 - 2742	1.4<br>98.6

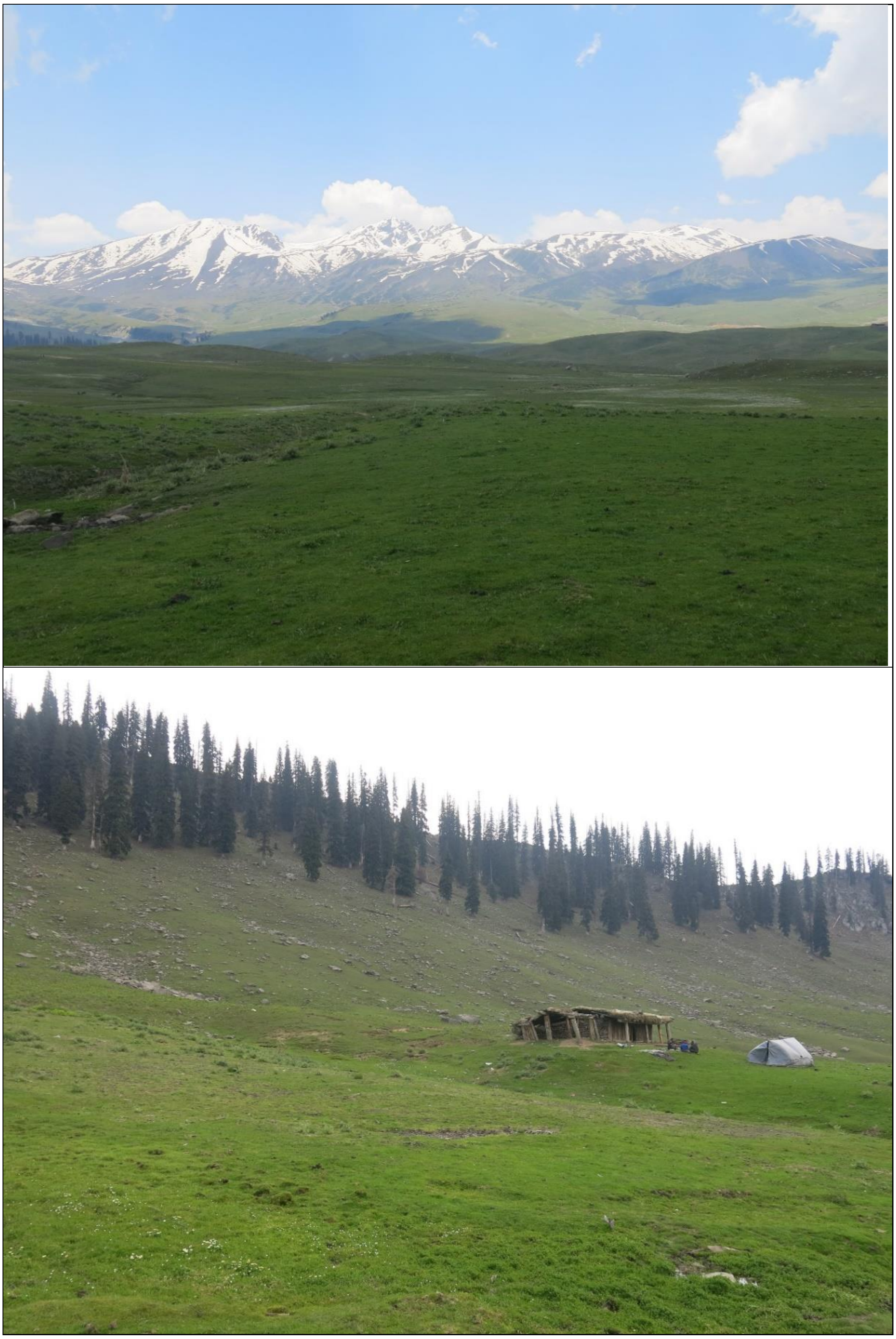

Figure S1: Tosa Maidan. Top – open meadow landscape; bottom – seasonal pastoralist habitation

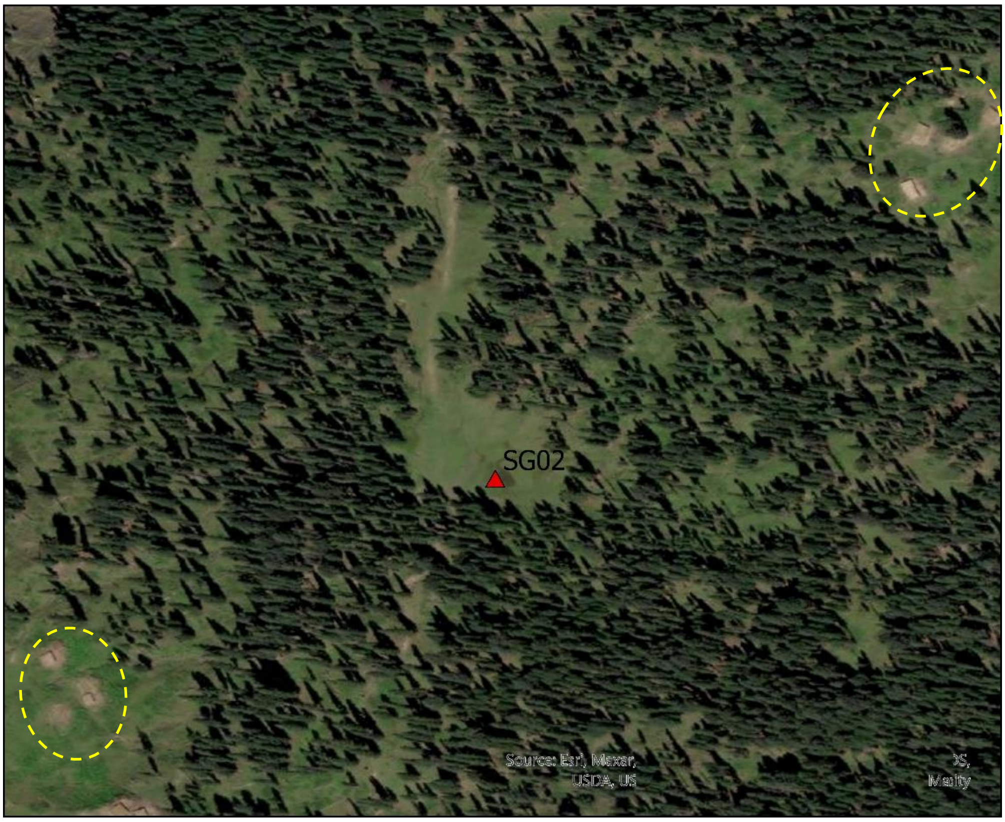

Figure S2: SG02 coring site showing location of forest openings and settlements (circled).

Source: ESRI world Imagery

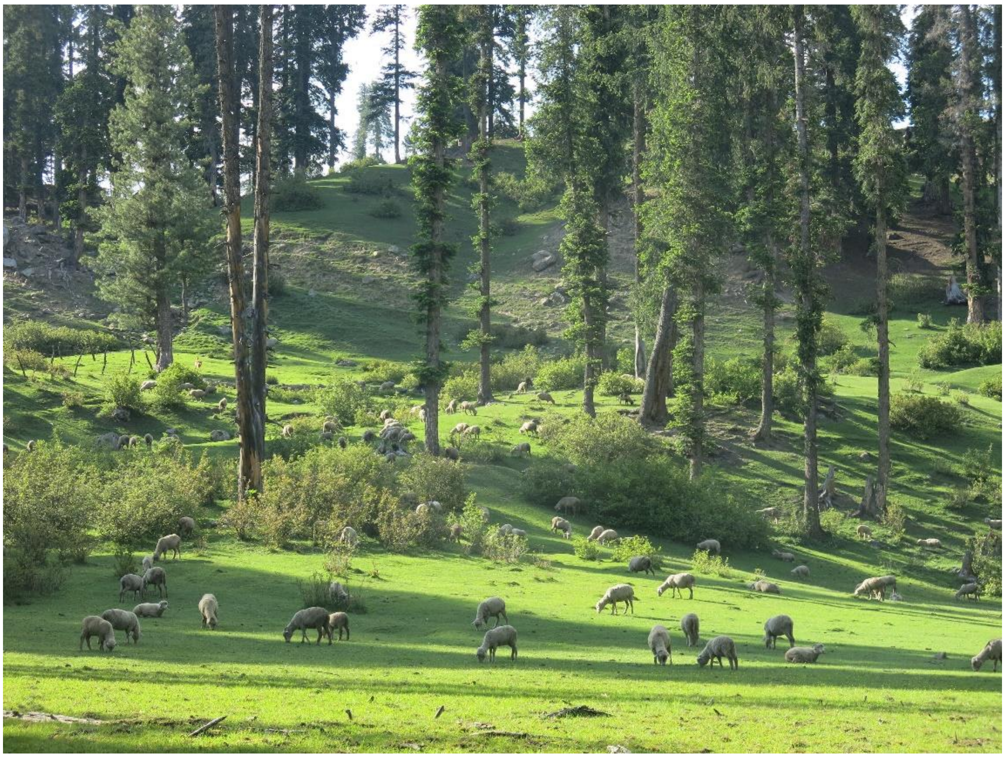

Figure S3: Present day land use, Pari Has. Summer 2018

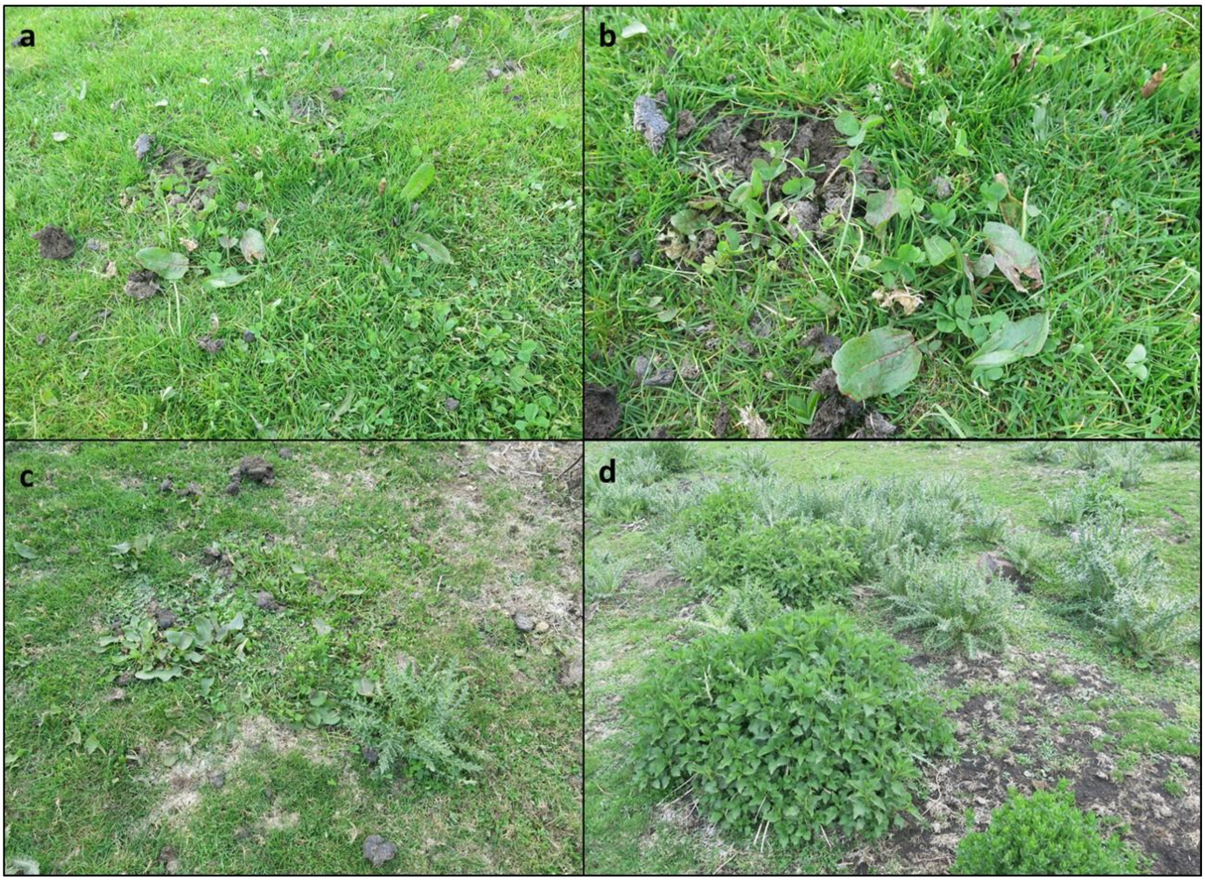

Figure S4: Impact of caprid grazing at Tosa Maidan. a & b - lightly grazed areas with dung accumulation, growth of *Rumex* and *Trifolium*, healthy groundcover; c - moderate grazing with evidence of trampling, dung accumulation, *Rumex* and thistle growth; d - Heavily grazed/degraded. Thistles and nettles dominate, surface erosion. (Summer 2018).

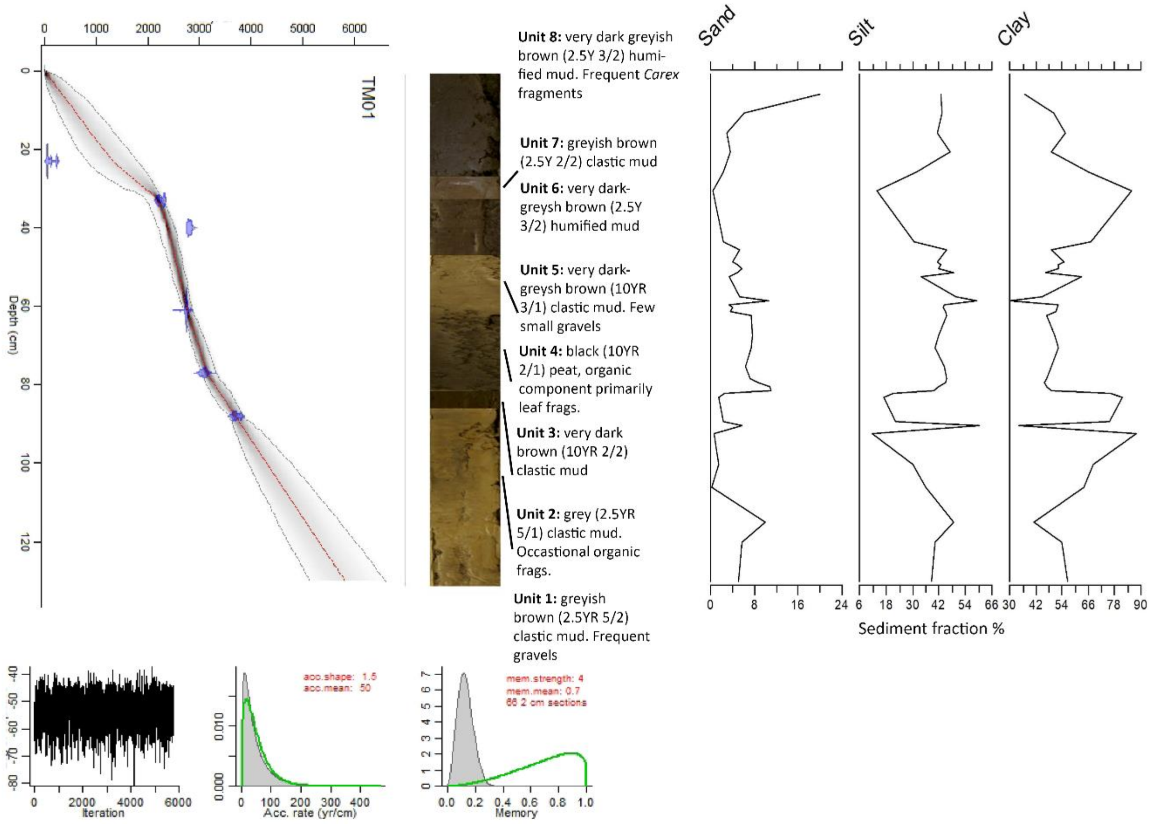

Figure S5: Age-depth model, graphical log and mineral sediment distribution plots TM01. TM01 is a core 130cm long and is composed of eight stratigraphic units. The age depth model was run in 66 sections of 2cm thickness and assumed a prior mean accumulation rate of 50 years/cm based on the overall length of the core (set by d.max=130). All other settings were left as default. Dates TM01_1.1_40 and TM01_1.1_23 fell outside of the models 95% confidence range. TM01_1.1_23 was unable to be calibrated and excluded by the model as a “modern” sample. TM01_1.1_40 was treated as a minor inversion and excluded by the model as an “outler”. A basal age of ca. 5500 BP was assumed based on modelled accumulation rates. Between ca. 5000-4000 BP sediment is dominated by clay, which we interpret elsewhere as a marker of cold-wet conditions [21]. Between 82-30cm overall, mean particle size increases to 3.4 ± 1.2µm, apparently relating to higher proportional inputs of sand (3.4-11%; mean 6.4 ± 2.4%). We interpret these changes as evidence of drier conditions as well as anthropogenically induced erosional processes resulting from land clearing and herding continuing until ca. 2500 BP. Following a decline, coarse sediments then increase sharply after ca. 1000 BP.

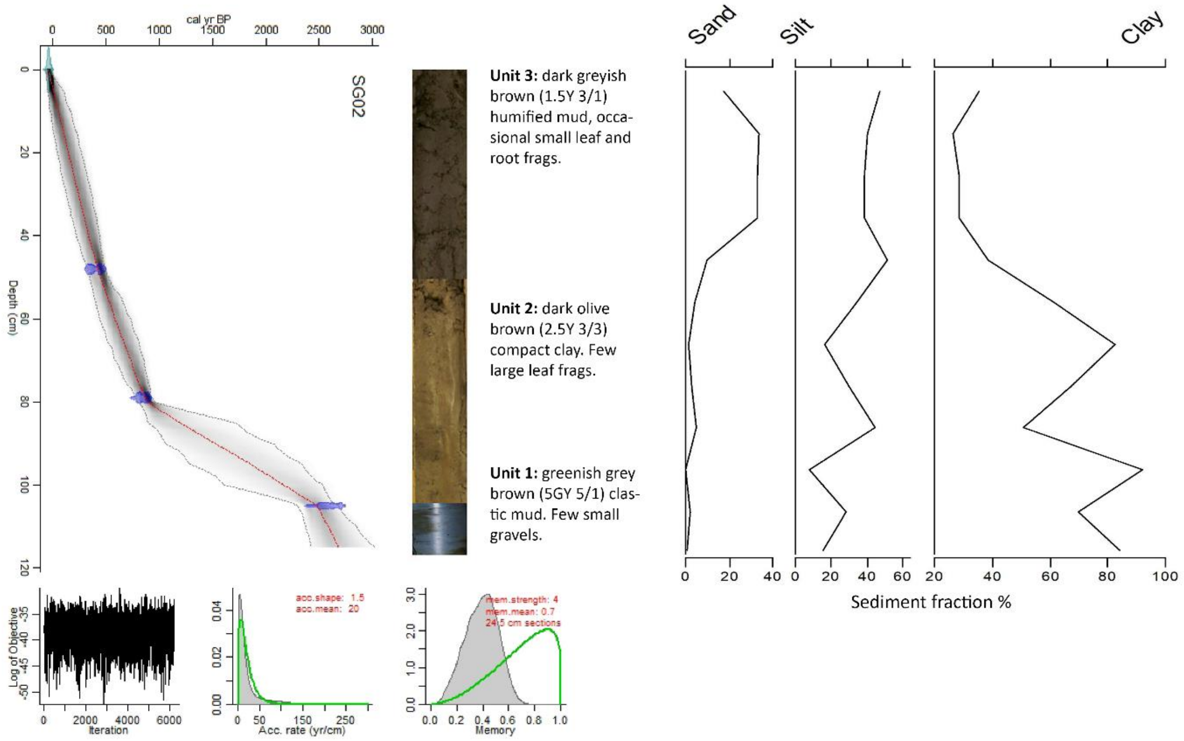

Figure S6: Age-depth model, graphical log and mineral sediment distribution plots SG02. SG02 is 115cm long core with three stratigraphic units. The Bacon age-depth model assumed a mean accumulation rate of 20yr/cm (based on d.max=115). The model was run in 24 sections of 5cm. All other settings were left as default. The model projects a basal age of the core to ca. 2700 BP. Modelled mean accumulation rates are generally low (<0.01cm/yr) between the dated materials at 79 and 105cm. This rate steadily and consistently climbs after 75cm, reaching a maximum rate of 0.2cm/yr towards the top of the core. This change in accumulation rate appears consistent with the stratigraphic changes from the initial core logging, with the more rapid accumulation rates correlated to sections with higher organic debris. Sediment particle size results are consistent with these stratigraphic changes, where between 115-55cm, clay is the dominant component, ranging from 55-92% (mean 75 ± 13.56%) of the distribution. Sand comprises 5% at 85cm and makes up no more than 1% of sediment elsewhere in this unit. Silt ranges between 8-45% (mean 23.5 ± 12%). Between 55-0cm, the sand fraction rises sharply to between 4.5-33% (mean 22 ± 11.9%) of the sediment size distribution. Clay has a mean of 36.3 ± 11.9% and silt averages 41.7 ± 5.89%. We interpret this influx of coarser sediments a result of anthropogenic impact on the landscape around the site after ca. 500 BP.

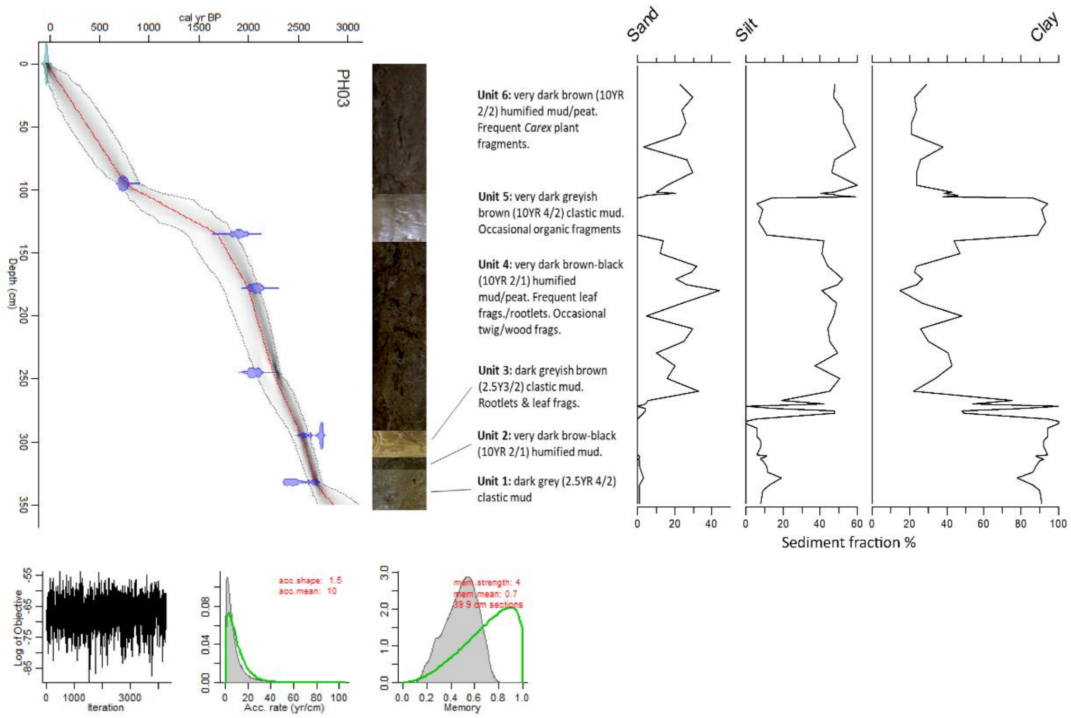

Figure S7: Age-depth model, graphical log and mineral sediment distribution plots PH03. Core PH03 is 355cm long and comprises six stratigraphic units. Age depth modelling assumed a prior average accumulation rate of 10 years/cm based on the oldest date distribution and length of the core (set by d.max=355). Running the model in thirty-nine 9cm thick sections allowed for all calibrated age distributions to fall within the model's 95% confidence range (329 years). Minor inversions between several dated samples introduced wider ranges of uncertainty within the model at some points. The maximum uncertainty was 707 years at 117cm depth. The modelled basal age was ca. 2800 BP. Sediment particle size was variable throughout, though four distinct sections can be clearly correlated with the stratigraphic units described during the initial logging. The largest variations relate to two minima in mean particle size at 100-130cm and 280-350cm. These minima are > 90% clay, with between 8 and 10% silt-sized clasts. Sand makes up no more than 4% of these minima and is generally in the range of 1-2%. These clay beds have been interpreted as wetter environmental phases between ca. 2750-2500 BP and 1700-900 BP, supported by higher proportions of marsh pollen types and fern spores [21]. Between 130-280cm, sand and silt average 17% and 37% of sediment composition. From 0-100cm sand comprises a mean of 21% and silt 51%, with a mean particle size of 11.5µm.

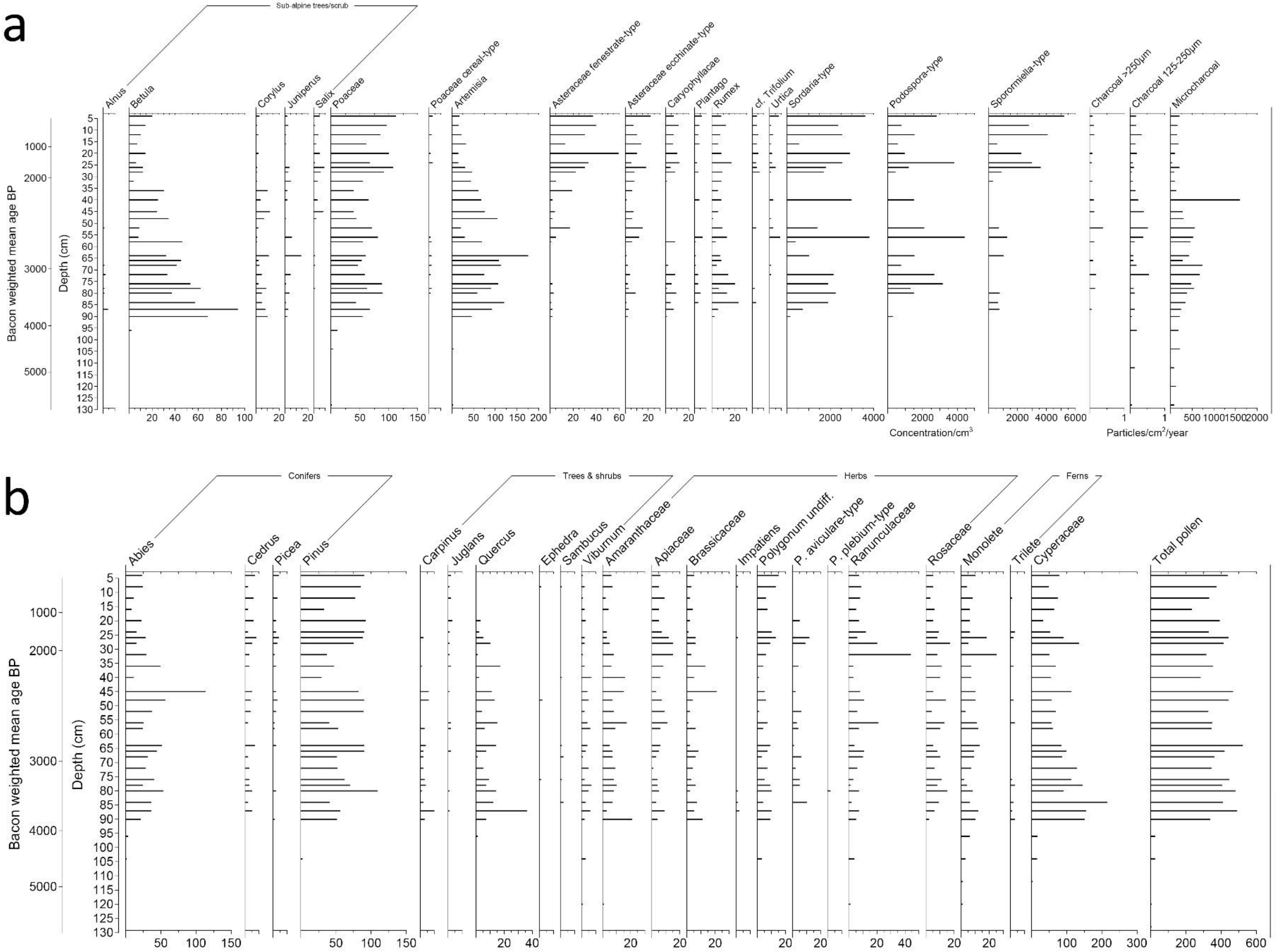

Figure S8: a) TM01 pollen absolute abundances, spore concentrations and charcoal influxes. These proxies were interpreted as relating to pastoralist activity around study site. We note declining sub-alpine trees/shrubs and *Artemisia* from ca. 3700BP. Variability in all other proxies is interpreted as discontinuous phases of grazing around the site.

b) absolute abundances of all other pollen and fern spores.

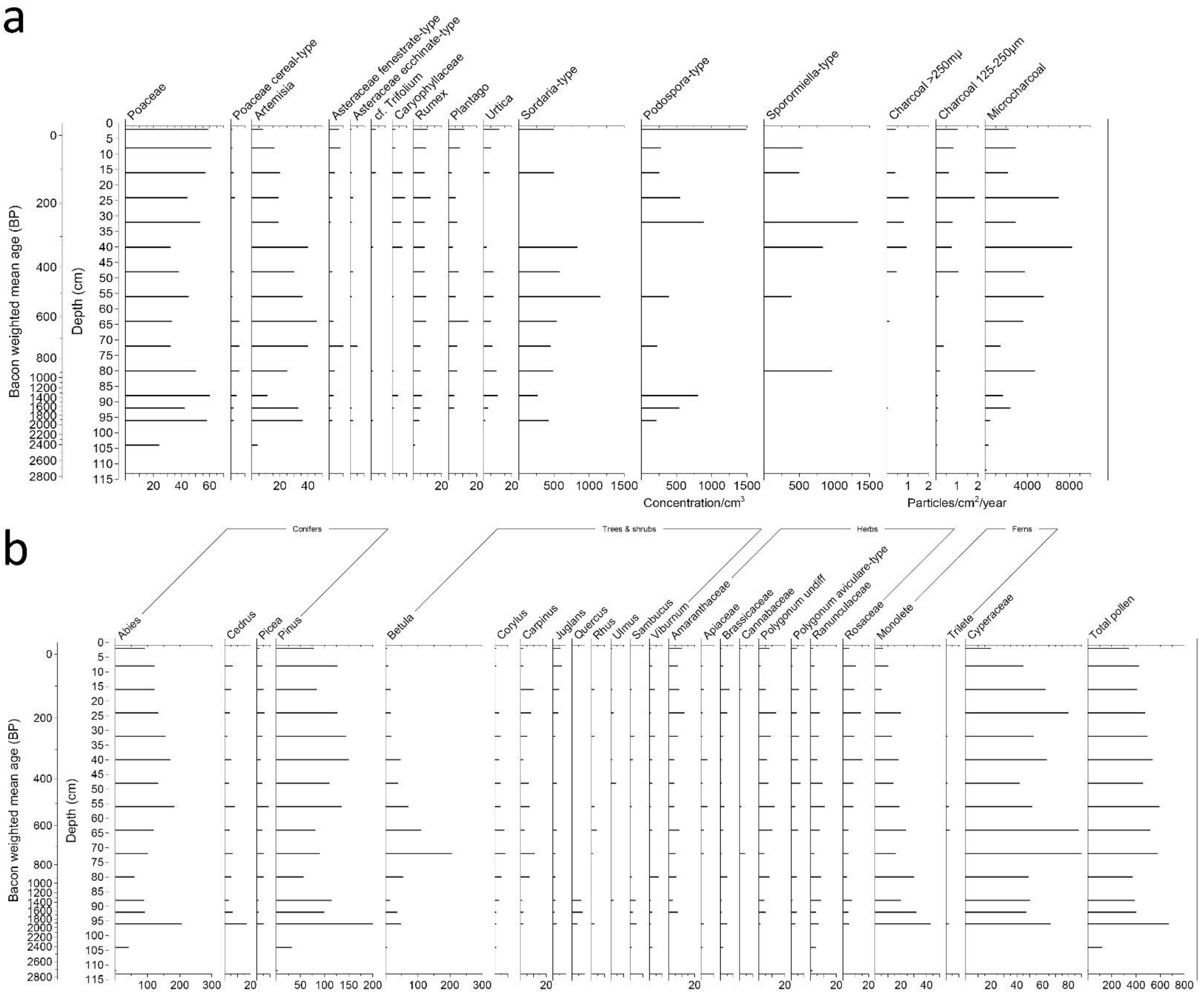

Figure S9: a) SG02 pollen absolute abundances, spore concentrations and charcoal influxes. We interpret these proxies as indicating intensifying land-use around the coring site seen in declining *Artemisia*, increasing Poaceae and coprophagous spores around the site after ca. 500 BP.

b) absolute abundances of all other pollen and fern spores.

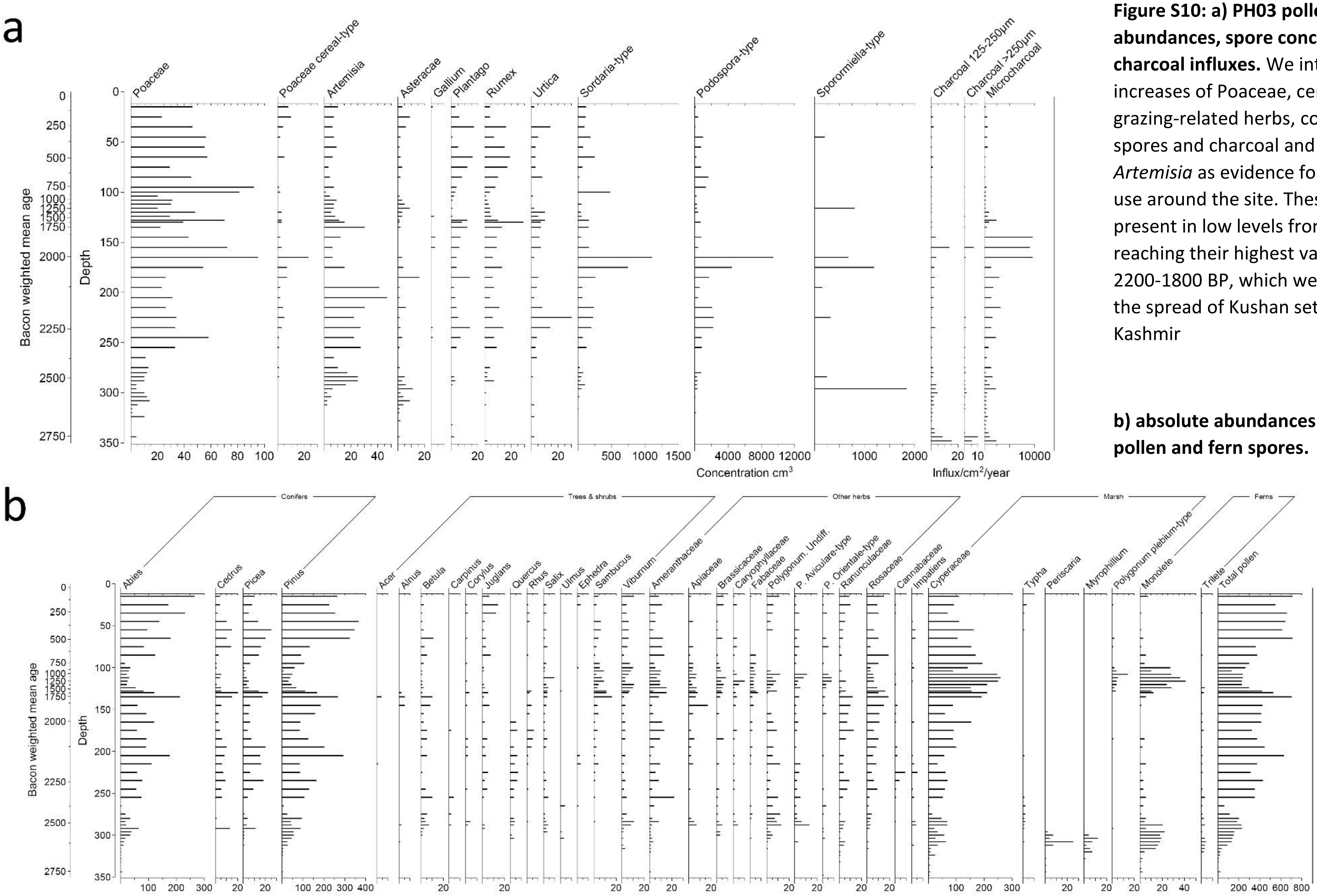

Figure S10: a) PH03 pollen absolute abundances, spore concentrations and charcoal influxes. We interpret the increases of Poaceae, cereal type pollens, grazing-related herbs, coprophagous spores and charcoal and decline in *Artemisia* as evidence for pastoralist land use around the site. These proxies are present in low levels from ca. 2700 BP, reaching their highest values between ca. 2200-1800 BP, which we associate with the spread of Kushan settlement in Kashmir

b) absolute abundances of all other pollen and fern spores.

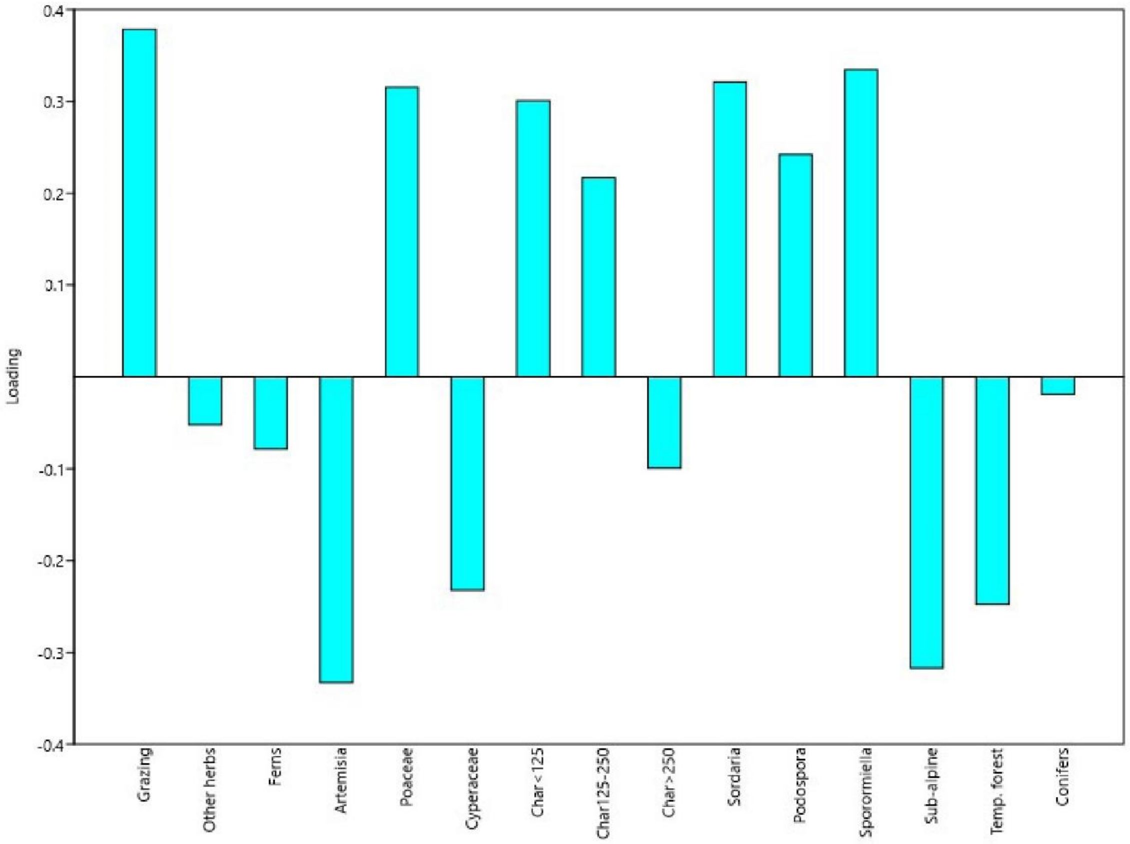

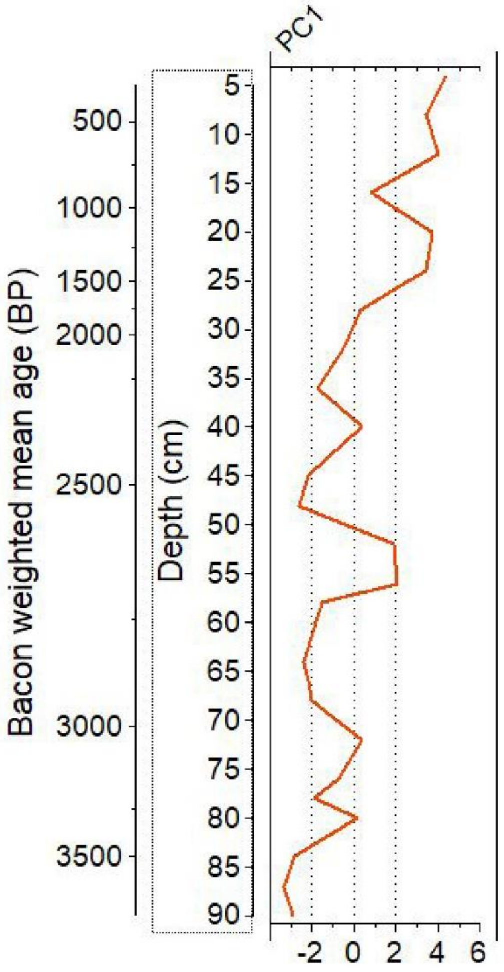

Figure S11: Top - PCA loading, core TM01, Principal component 1 explains 38% variance in the dataset. PC1 has strong positive loadings for variables that may be associated with pastoralist activity, including Poaceae (0.31), grazing related pollen types (0.38) and coprophagous spore types (0.24-0.33). Negative loadings on PC1 include sub-alpine trees/shrubs (-0.31) and *Artemisia* (-0.33). As high altitude *Betula* was the primary taxa in the tree/shrub grouping, both variables may be interpreted as land cover that is particularly susceptible to land clearing and grazing. This exploration of variance among the data supports our interpretation regarding proxies indicating pastoralist land use in the TM01 record.

Bottom – Plot of PC1 scores reflecting higher values for pastoralist land use ca. 3500-3000 BP, 2700-2500 BP and 2000 BP- present.

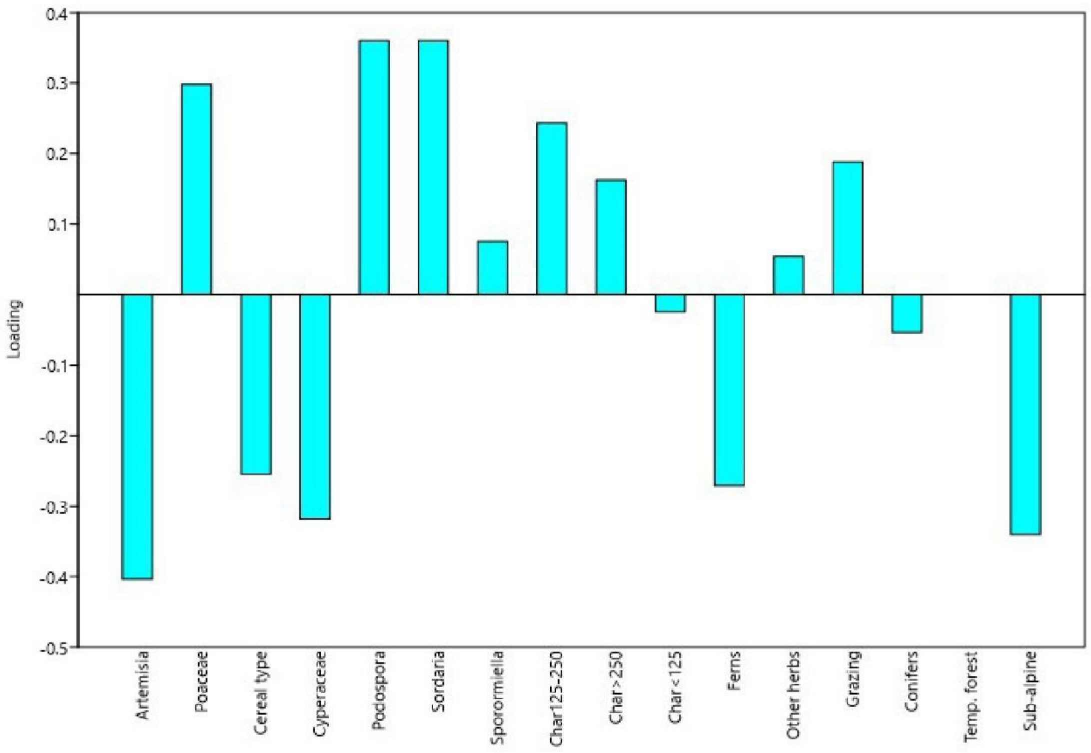

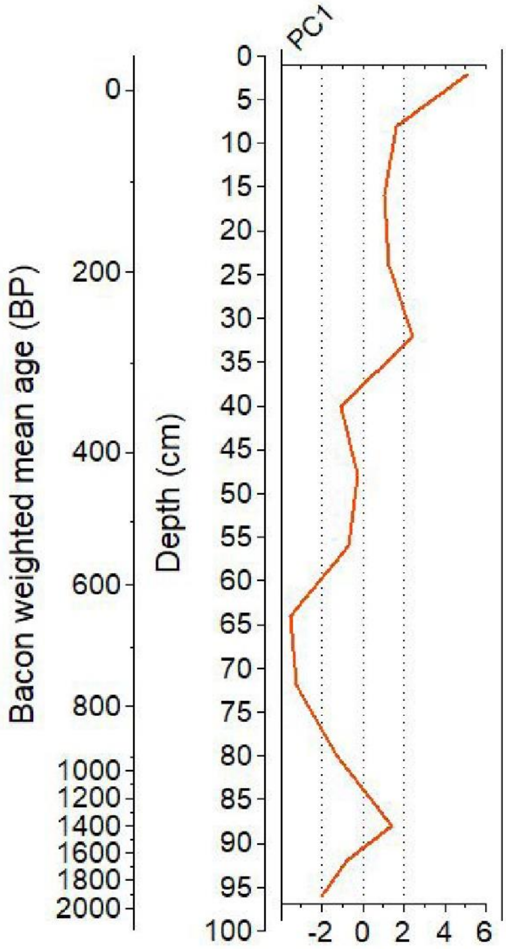

Figure S12: Top- PCA loading, core SG02, Principal component 1 explains 32% variance in the dataset. This axis has strong positive loadings for Poaceae (0.29), Podospora-type and Sordaria-type spores (0.36 each) and grazing related herbs (0.18). PC1 also has strong negative values for *Artemisia* (−0.4) as well as sub-alpine and conifer tree pollens. The antagonistic relationship between these variables indicates that that on PC1 is likely reflects intensified grazing activity, supporting our interpretation of proxies from core SG02.

Bottom – Plot of PC1 scores reflecting intensified pastoralist activity around site after ca. 500 BP.

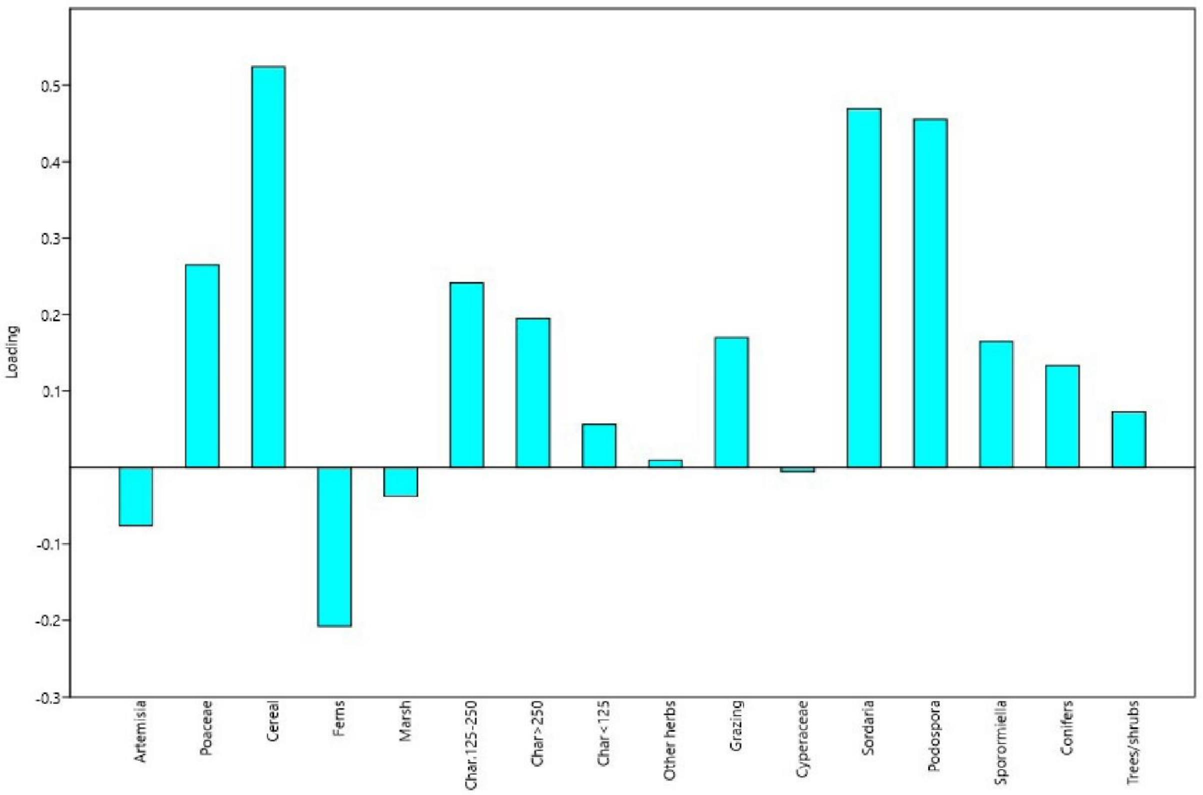

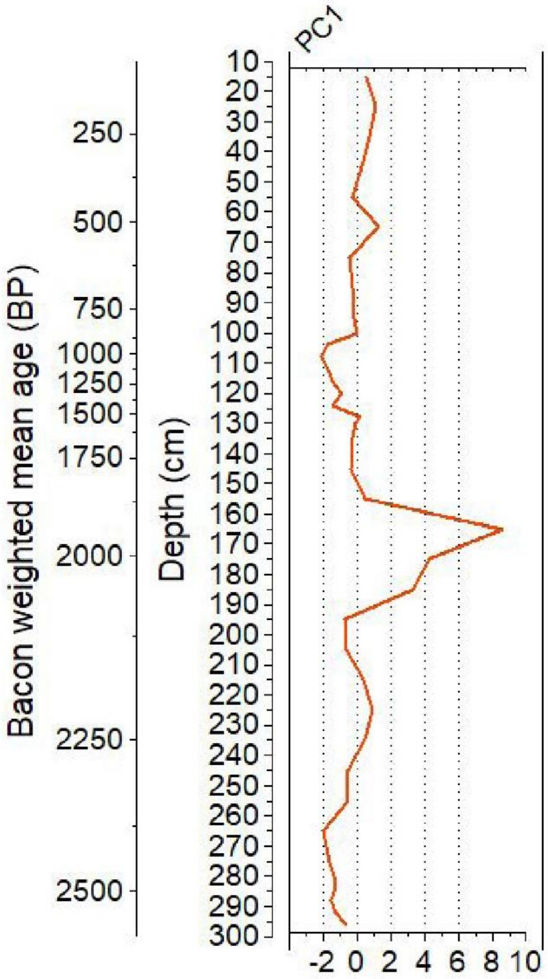

Figure S13: Top - PCA loading, core PH03, Principal component 1 explains 24.5% of variation among the data. Positive loadings of Poaceae (0.26), cereal-type (0.52) and grazing related pollen (0.16) as well for all charcoal size classes and coprophagous spore types allow us to interpret these data are indicative of agro-pastoralist activity around the study site.

Bottom – Plot of PC1 scores reflecting phases of intensified grazing and cultivation around site ca. 2400-2200 BP, sharply increasing ca. 2100-1800 BP then declining until ca. 800 BP-present.

TM01

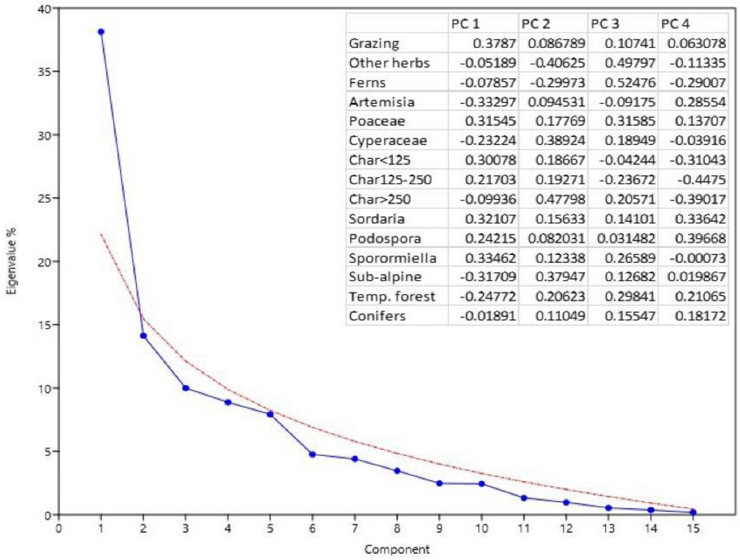

	PC 1	PC 2	PC 3	PC 4
Grazing	0.3787	0.086789	0.10741	0.063078
Other herbs	-0.05189	-0.40625	0.49797	-0.11335
Ferns	-0.07857	-0.29973	0.52476	-0.29007
Artemisia	-0.33297	0.094531	-0.09175	0.28554
Poaceae	0.31545	0.17769	0.31585	0.13707
Cyperaceae	-0.23224	0.38924	0.18949	-0.03916
Char<125	0.30078	0.18667	-0.04244	-0.31043
Char125-250	0.21703	0.19271	-0.23672	-0.4475
Char>250	-0.09936	0.47798	0.20571	-0.39017
Sordaria	0.32107	0.15633	0.14101	0.33642
Podospora	0.24215	0.082031	0.031482	0.39668
Sporormiella	0.33462	0.12338	0.26589	-0.00073
Sub-alpine	-0.31709	0.37947	0.12682	0.019867
Temp. forest	-0.24772	0.20623	0.29841	0.21065
Conifers	-0.01891	0.11049	0.15547	0.18172

SG02

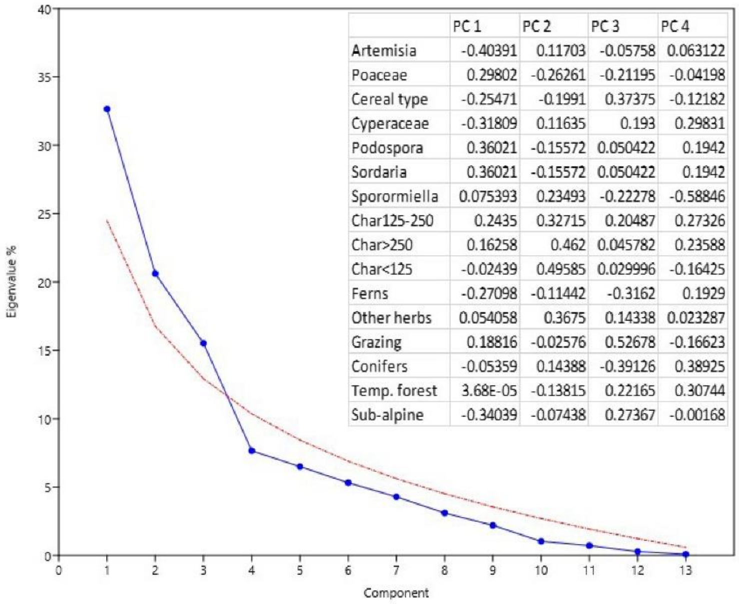

	PC 1	PC 2	PC 3	PC 4
Artemisia	-0.40391	0.11703	-0.05758	0.063122
Poaceae	0.29802	-0.26261	-0.21195	-0.04198
Cereal type	-0.25471	-0.1991	0.37375	-0.12182
Cyperaceae	-0.31809	0.11635	0.193	0.29831
Podospora	0.36021	-0.15572	0.050422	0.1942
Sordaria	0.36021	-0.15572	0.050422	0.1942
Sporormiella	0.075393	0.23493	-0.22278	-0.58846
Char125-250	0.2435	0.32715	0.20487	0.27326
Char>250	0.16258	0.462	0.045782	0.23588
Char<125	-0.02439	0.49585	0.029996	-0.16425
Ferns	-0.27098	-0.11442	-0.3162	0.1929
Other herbs	0.054058	0.3675	0.14338	0.023287
Grazing	0.18816	-0.02576	0.52678	-0.16623
Conifers	-0.05359	0.14388	-0.39126	0.38925
Temp. forest	3.68E-05	-0.13815	0.22165	0.30744
Sub-alpine	-0.34039	-0.07438	0.27367	-0.00168

Figure S14: Scree plot with broken stick and PC scores for first 4 components. Components 2-4 interpreted as either not significant (TM01) or not relevant to present study (SG02 & PH03).

PH03

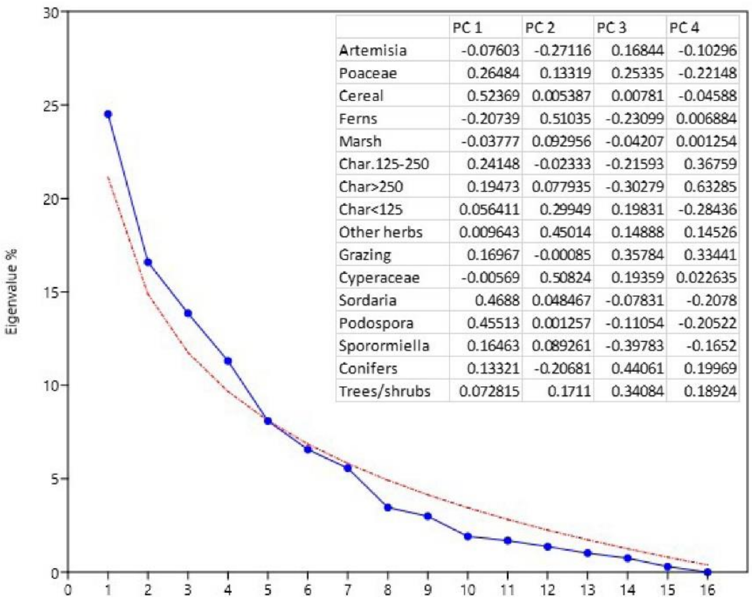

	PC 1	PC 2	PC 3	PC 4
Artemisia	-0.07603	-0.27116	0.16844	-0.10296
Poaceae	0.26484	0.13319	0.25335	-0.22148
Cereal	0.52369	0.005387	0.00781	-0.04588
Ferns	-0.20739	0.51035	-0.23099	0.006884
Marsh	-0.03777	0.092956	-0.04207	0.001254
Char.125-250	0.24148	-0.02333	-0.21593	0.36759
Char>250	0.19473	0.077935	-0.30279	0.63285
Char<125	0.056411	0.29949	0.19831	-0.28436
Other herbs	0.009643	0.45014	0.14888	0.14526
Grazing	0.16967	-0.00085	0.35784	0.33441
Cyperaceae	-0.00569	0.50824	0.19359	0.022635
Sordaria	0.4688	0.048467	-0.07831	-0.2078
Podospora	0.45513	0.001257	-0.11054	-0.20522
Sporormiella	0.16463	0.089261	-0.39783	-0.1652
Conifers	0.13321	-0.20681	0.44061	0.19969
Trees/shrubs	0.072815	0.1711	0.34084	0.18924

Supplement References

1. Pearsall, D. M. *Paleoethnobotany: a handbook of procedures*. (Left Coast Press Inc, 2015).
2. Seppä, H. POLLEN ANALYSIS, PRINCIPLES. in *Encyclopedia of Quaternary Science* 2486–2497 (Elsevier, 2007). doi:10.1016/B0-44-452747-8/00175-7.
3. Singh, G. A Preliminary Survey of the Post-Glacial Vegetational History of the Kashmir Valley. *The Palaeobotanist* **12**, 73–108 (1963).
4. Singh, G. & Agrawal, D. P. Radiocarbon evidence for deglaciation in north-western Himalaya, India. *Nature* **260**, 232–232 (1976).
5. Stein, M. A. *Memoir on Maps Ilustrating the Ancient Geography of Kashmir*. (Journal of the Royal Asiatic Society of Bengal, 1899).
6. Casimir, M. J. & Rao, A. Vertical Control in the Western Himalaya: Some Notes on the Pastoral Ecology of the Nomadic Bakrwal of Jammu and Kashmir. *Mt. Res. Dev.* **5**, 221–232 (1985).
7. Quamar, M. F. & Kar, R. Modern pollen dispersal studies in India: a detailed synthesis and review. *Palynology* **44**, 217–236 (2020).
8. Vishnu-Mittre. Some aspects of concerning pollen analytical investigations in the Kashmir valley. *The Palaeobotanist* **15**, 157–175 (1966).
9. Ahmad, S., Dar, H. U., Dar, J. A. & Majeedi, Z. M. Impact of varying disturbances on the structure and composition of grassland vegetation in Anantnag, Kashmir Himalayas. *Proc. Int. Acad. Ecol. Environ. Sci.* (2013).
10. Dad, J. . M. & Khan, A. . B. Floristic composition of an alpine grassland in Bandipora, Kashmir. *Grassl. Sci.* **56**, 87–94 (2010).
11. Dad, J. . M. & Reshi, Z. A. Classification and Compositional Differentiation of Alpine Grassland Vegetation of Gurez Valley, Kashmir. *Asian J. Biol. Sci.* **6**, 192–206 (2013).
12. Khuroo, A. A. FLORISTIC DIVERSITY OF KASHMIR HIMALAYAN GRASSLANDS IN RELATION TO THEIR FUNCTIONING. (Univeristy of Kashmir, 2013).

13. Mir, F. A., Bhat, G. A., Sheer, B. A. & Khan, A. G. IMPACTS OF SEASONAL LIVESTOCK GRAZING ON PLANT COMMUNITY FEATURES: A CASE STUDY ON GRAZING PASTURES AT SONAMARGAREA IN KASHMIR HIMALAYA. *J. Agric. Sci.* **10**, 41–48 (2015).
14. Jaweed, T. H., Saptarshi, P. G. & Gaikwad, S. W. Impact of transhumant grazing on physical and chemical properties of soils in temperate pasturelands of Kashmir Himalaya. *Range Manag. Agrofor.* **36**, 128–135 (2015).
15. Mooney, S. D. & Tinner, W. The analysis of charcoal in peat and organic sediments. *Mires Peat* **7**, 1–18 (2011).
16. Basumatary, S. K. *et al.* Coprophilous and non-coprophilous fungal spores of Bos mutus modern dung from the Indian Himalaya: Implications to temperate paleoherbivory and paleoecological analysis. *Rev. Palaeobot. Palynol.* **277**, 104208 (2020).
17. Spengler, R. N. Niche Dwelling vs. Niche Construction: Landscape Modification in the Bronze and Iron Ages of Central Asia. *Hum. Ecol.* **42**, 813–821 (2014).
18. van Asperen, E. N., Kirby, J. R. & Shaw, H. E. Relating dung fungal spore influx rates to animal density in a temperate environment: Implications for palaeoecological studies. *The Holocene* 095968361987580 (2019) doi:10.1177/0959683619875804.
19. Reimer, P. J. *et al.* The IntCal20 Northern Hemisphere Radiocarbon Age Calibration Curve (0–55 cal kBP). *Radiocarbon* **62**, 725–757 (2020).
20. Stuvier, M., Reimer, R. W. & Reimer, P. J. *Calib*. (2021).
21. Spate, M., Penny, D., Yatoo, M. & Betts, A. Re-examining climate-driven Malthusian collapse in Kashmir: New palaeoenvironmental context for the archaeological record. *Quat. Int.* S1040618221004079 (2021) doi:10.1016/j.quaint.2021.07.014.
